# Supplementary material for: Gene expression noise can promote the fixation of beneficial mutations in fluctuating environments
Source: PLoS Comput Biol. 2020 Oct 26;16(10):e1007727. doi: 10.1371/journal.pcbi.1007727 (PMC7644098; doi:10.1371/journal.pcbi.1007727)
Supplement: S1 Appendix — This appendix shows parameter values, variables, and further details about the model and its implementation. It presents an analysis of how a growing and a nongrowing state can arise in a deterministic version of the model, and estimates how robust this bistability is against changes in parameter values. It also considers how Cra amounts influence the growth state a cell is in. In addition, it contains simulations showing the effect of active degradation of the acetate incorporation enzyme. Finally, it considers the spread of a neutral allele in populations with different levels of Cra expression noise and concludes with simulations of randomly fluctuating environments. (PDF) [file pcbi.1007727.s001.pdf]

Supplementary appendix S1 to ‘Gene expression noise can promote the fixation of beneficial mutations in fluctuating environments’

Contents

1 Table of parameters **2**

2 Table of variables **3**

3 Cra expression noise: Parameter values **3**

4 Code implementation **3**

5 Additional model details **4**

5.1 Extracellular metabolite concentrations 4

5.2 Reaction rates 4

5.3 Cell efflux 5

6 A deterministic version of the model is bistable **5**

7 Searching parameter space: How likely is bistability, and what influences it? **13**

8 The number of Cra proteins affects the probability of growth on acetate **16**

9 Protein degradation does not remove the fitness benefit of noise when Cra-fbp binding is tight **17**

10 Invasion probabilities of a neutral allele **21**

11 The benefits of Cra expression noise are less pronounced in environments that fluctuate randomly **23**

1 Table of parameters

Parameter	Value	Interpretation
V	1×10^{-9} L	Volume of the growth environment
D	0.1 h^{-1}	Dilution rate
B_0	3×10^{-13}g	Mass of a newborn cell (dry weight)
$K_{Gi,glc}$	0.1 mmol L^{-1}	MM dissociation constant
$K_{Ai,ac}$	7.0 mmol L^{-1}	MM dissociation constant
$K_{Lg,pep}$	0.3 mmol g^{-1}	MM dissociation constant
$K_{Cra,fbp}$	0.1 mmol g^{-1}	Hill dissociation constant
$K_{Cra_A,DNA}$	25 $cell^{-1}$	Promoter dissociation constant
$K_{An,fbp}$	0.1 mmol g^{-1}	MWC dissociation constant
$K_{An,pep}$	0.008 mmol g^{-1}	MWC dissociation constant
L_{An}	4×10^6	MWC allosteric constant
n_{Cra}	2	Cra-fbp Hill coefficient
n_{fbp}	4	MWC cooperativity constant
c	0.0896 g $mmol^{-1}$	Conversion factor fbp to biomass
$kcat_{Gi,s}$	100 s^{-1}	Turnover rate
$kcat_{Ai,s}$	100 s^{-1}	Turnover rate
$kcat_{Lg,s}$	200 s^{-1}	Turnover rate
$kcat_{An,s}$	200 s^{-1}	Turnover rate
α_{Gi}	10 per protein half-life	Gi mRNA production rate
$\alpha_{Ai,0}$	0 per protein half-life	Ai mRNA production rate
$\alpha_{Ai,1}$	50 per protein half-life	Ai mRNA production rate
α_{Lg}	20 per protein half-life	Lg mRNA production rate
α_{An}	10 per protein half-life	An mRNA production rate
α_{Cra}	5 per protein half-life	Cra mRNA production rate
β_{Gi}	500 per mRNA	Gi protein burst size
β_{Ai}	400 per mRNA	Ai protein burst size
β_{Lg}	200 per mRNA	Lg protein burst size
β_{An}	200 per mRNA	An protein burst size
β_{Cra}	20 per mRNA	Cra protein burst size
γ_p	0 h^{-1}	Ai degradation rate

2 Table of variables

Variable	Units	Interpretation
N	∅	Number of cells
glc	mM	Extracellular glucose concentration
glc_{in}	mM	Concentration of inflowing glucose
glc_{uptake}	$\mathrm{mM\,h^{-1}}$	Rate of glucose assimilation
ac	mM	Extracellular acetate concentration
ac_{in}	mM	Concentration of inflowing acetate
ac_{uptake}	$\mathrm{mM\,h^{-1}}$	Rate of acetate assimilation
pep	$\mathrm{mmol\,g^{-1}}$	Intracellular concentration
fbp	$\mathrm{mmol\,g^{-1}}$	Intracellular concentration
B	g	Cell mass (dry weight)
J_{Gi}	$\mathrm{mmol\,g^{-1}\,h^{-1}}$	Glucose influx
J_{Ai}	$\mathrm{mmol\,g^{-1}\,h^{-1}}$	Acetate influx
J_{Lg}	$\mathrm{mmol\,g^{-1}\,h^{-1}}$	pep consumption flux
J_{An}	$\mathrm{mmol\,g^{-1}\,h^{-1}}$	fbp consumption flux
μ	$\mathrm{h^{-1}}$	Specific growth rate
Gi	∅	Protein number
Ai	∅	Protein number
Lg	∅	Protein number
An	∅	Protein number
Cra	∅	Protein number
Cra_A	∅	Cra activity
α_{Ai}	per protein half-life	Ai mRNA production rate
γ_d	$\mathrm{h^{-1}}$	Protein dilution rate
k_1	$\mathrm{h^{-1}}$	Transcription rate

3 Cra expression noise: Parameter values

Noise (η^2)	α_{Cra}	β_{Cra}
0.01	100	1
0.1	10	10
0.2	5	20
1.0	1	100
10.0	0.1	1000

4 Code implementation

We performed all modelling in python 2.7. During simulations, we observed that excessively small concentrations could lead to negative concentration values of some metabolites. To avoid negative concentrations, we set any metabolite concentration to zero if there is less than 1 molecule either in the growth environment or within a given cell. To calculate the number of molecules for any one molecular species in a cell, the concentration of these molecules is multiplied with the cell mass for intracellular metabolites or with the volume of the environment for extracellular metabolites, and divided by Avogadro's number. We performed all statistical analyses in R 3.2.3 [1]. All figures are plotted in matplotlib 2.1.0 [2].

5 Additional model details

In this part we describe details of the system of ordinary differential equations that model the change in both extracellular and intracellular metabolite concentrations, given the rates of the metabolic reactions occurring in every cell. We also discuss the process of cell efflux, where cells are flushed out of the growth environment at random.

5.1 Extracellular metabolite concentrations

We simulate growth in an environment with constant flow of medium entering and leaving the system at a “dilution” rate D h^{-1}. The concentrations of glucose and acetate in the incoming medium are denoted by glc_{in} and ac_{in} mM respectively. A population of cells takes up these carbon source at rates glc_{uptake} and ac_{uptake} mM h^{-1}, respectively. These considerations give rise to two ODEs for the concentrations of glucose glc and acetate ac in the environment (in mM).

$$\frac{\mathrm{d}glc}{\mathrm{d}t} = glc_{in} \cdot D - glc_{uptake} - glc \cdot D \tag{1}$$

$$\frac{\mathrm{d}ac}{\mathrm{d}t} = ac_{in} \cdot D - ac_{uptake} - ac \cdot D \tag{2}$$

Because the uptake rates of glucose and acetate, J_{Gi} and J_{Ai}, vary between cells, we obtain the overall rate of a population's carbon source consumption by summing over all N individuals in the population. Because intracellular metabolite concentrations are given per unit biomass, to calculate the change in concentration in the growth environment we need to convert the the concentrations of the intracellular metabolites in mmol g^{-1} h^{-1} to those of the extracellular metabolites in mM h^{-1} using the biomass of each cell B_i and the volume of the growth environment V. In this way, we arrive at the following expressions for both carbon source uptake rates

$$glc_{uptake} = \frac{1}{V} \sum_{i=1}^{N} J_{Gi,i} \cdot B_i \tag{3}$$

$$ac_{uptake} = \frac{1}{V} \sum_{i=1}^{N} J_{Ai,i} \cdot B_i \tag{4}$$

5.2 Reaction rates

The rate of each chemical reaction is calculated for each cell using the intra- and extracellular concentrations of the relevant metabolites, the number of enzymes catalysing the reaction in the cell, as well as the cell mass at any given point in time. The turnover number of every enzyme $kcat$ is converted from

s^{-1} to mmol h^{-1} using equation 5 [3].

$$kcat = \frac{kcat_s \cdot 3600}{6.023 \times 10^{20}} \tag{5}$$

because there are 3600 s h^{-1} and 6.023×10^{20} $mmol^{-1}$. All enzymatic steps follow Michaelis-Menten kinetics except for anabolism An, which converts fbp into biomass and follows Monod-Wyman-Changeux kinetics [4]. All reaction rates are divided by the current cell mass B to account for an enzyme's dilution during cell growth. In sum, reaction fluxes are given by

$$J_{Gi} = \frac{kcat_{Gi} \cdot Gi}{B} \cdot \frac{glc}{K_{Gi,glc} + glc} \tag{6}$$

$$J_{Ai} = \frac{kcat_{Ai} \cdot Ai}{B} \cdot \frac{ac}{K_{Ai,ac} + ac} \tag{7}$$

$$J_{Lg} = \frac{kcat_{Lg} \cdot Lg}{B} \cdot \frac{pep}{K_{Lg,pep} + pep} \tag{8}$$

$$J_{An} = \frac{kcat_{An} \cdot An}{B} \cdot \frac{\frac{fbp}{K_{An,fbp}} \cdot (1 + \frac{fbp}{K_{An,fbp}})^{n_{fbp}-1}}{(1 + \frac{fbp}{K_{An,fbp}})^{n_{fbp}} + L_{An} \cdot (1 + \frac{pep}{K_{An,pep}})^{-n_{fbp}}} \tag{9}$$

Because both protein and metabolite concentrations are given per unit mass (g^{-1}), metabolite fluxes are given in mmol g^{-1} h^{-1}.

5.3 Cell efflux

A constant flow of nutrients through the simulated growth environment also leads to cells being flushed out at random, a process that we model stochastically as follows. Given an initial populations size of N_0 cells, the number of survivors after a given time interval Δt follows a binomial distribution

$$N_t \sim Bin(N_0, p) \tag{10}$$

where p is the probability of survival and decays exponentially over time at a constant rate set by the dilution rate D

$$p = e^{-Dt}. \tag{11}$$

6 A deterministic version of the model is bistable

In this section, we use a combination of analytical and numerical approaches to confirm that our model generates two stable states on acetate that correspond to a growing and a nongrowing subpopulation. We use the term stability here in the sense that once metabolism settles in a given growth state, it remains there unless perturbed from outside and will return to that state if perturbed. The following

analysis also allows us to show precisely how the components of our model interact to form this bistable behaviour. In order to do so, we use a simpler, deterministic variant of our cell growth model.

This deterministic model differs from the stochastic model in two important ways. First, we remove protein expression noise, i.e. proteins are always present at exactly their mean number per cell. Because we have made the simplifying assumption in our stochastic model that the average number of constitutively expressed proteins per unit biomass is constant, we here assume that the concentrations of these proteins are fixed and treat them as constants in our deterministic model. We do so for all proteins except for the acetate incorporation enzyme Ai, whose expression depends on Cra activity. To make the analysis easier, we treat Ai as a continuous variable, namely as the concentration of Ai per unit mass. As explained in the main text, Ai is produced at a rate determined by the fbp concentration as mediated by Cra activity. We summarise this process in a single function that specifies the Ai production rate given the fbp concentration, $P_{Ai}(fbp)$. For simplicity, we assume that Ai is not degraded (although its concentration can diminish during cell growth). Second, because all variables are now concentrations given per unit biomass, we no longer need to model biomass growth explicitly. Instead, our simplified model system only needs to take into account that the acetate incorporation enzyme Ai and the intracellular metabolites pep and fbp can get diluted through cell growth.

To show how reaction rate J_x depends on the concentration of its substrate x, and in the case of acetate uptake on the concentration of the acetate incorporation protein Ai, we will denote all reaction rates as functions. Thus, the rates of acetate incorporation, lower glycolysis, and anabolism are given as $J_{Ai}(Ai, ac)$, $J_{Lg}(pep)$ and $J_{An}(pep, fbp)$ respectively. Also, because here we are especially interested in cell growth on acetate as the only available carbon source, we can drop the term for the rate of glucose influx J_{Gi}. Consequently, our deterministic model contains only three ordinary differential equations (ODEs)

$$\begin{aligned}
\frac{\mathrm{d}pep}{\mathrm{d}t} &= \frac{1}{2}J_{Ai}(Ai, ac) - J_{Lg}(pep) - c \cdot J_{An}(pep, fbp) \cdot pep \\
\frac{\mathrm{d}fbp}{\mathrm{d}t} &= \frac{1}{2}J_{Lg}(pep) - J_{An}(pep, fbp) - c \cdot J_{An}(pep, fbp) \cdot fbp \\
\frac{\mathrm{d}Ai}{\mathrm{d}t} &= c \cdot J_{An}(pep, fbp) \cdot (P_{Ai}(fbp) - Ai)
\end{aligned} \tag{12}$$

where c is a conversion factor between the anabolism reaction rate J_{An} and the growth rate μ (see Materials and Methods in main text). The ODE system contains only three variables, namely the intracellular concentrations pep and fbp, and the concentration of the acetate incorporation protein Ai. As we show below, the external acetate concentration ac plays an important role in determining the equilibrium behaviour of this ODE system.

We start the analysis by finding out where the ODE system is at equilibrium, that is, for which values of the three variables (and given the extracellular concentration of acetate) there is no change in the

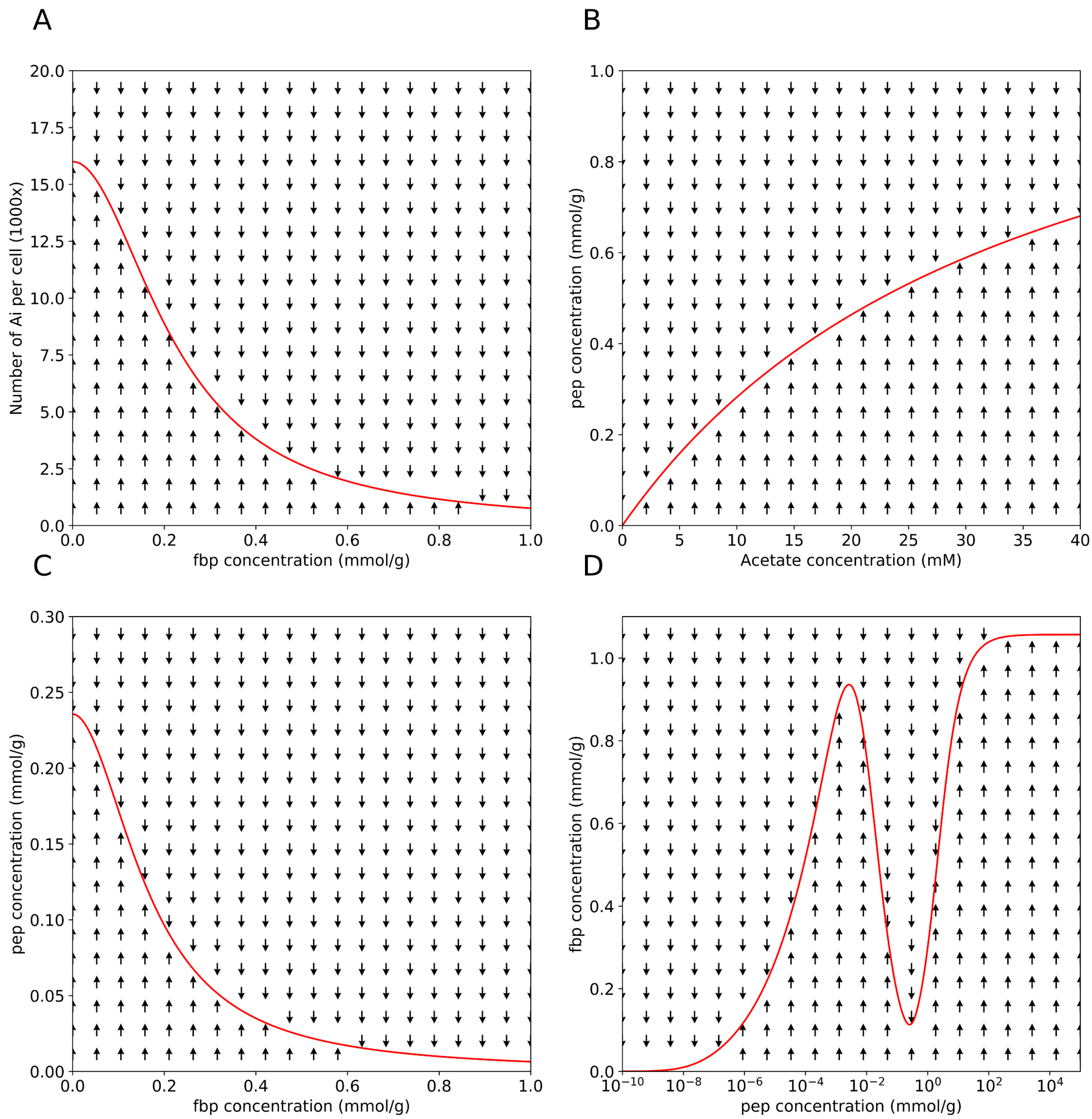

Figure 1: Behaviour of the three equations in the deterministic model (ODE system 12), with each equation considered in isolation with respect to its variables. In every panel, the vertical axis shows the variable whose rate of change is determined by the differential equation examined in that panel, while the horizontal axis shows one of the other variables that affects the equation in question. All other variables are held constant. Arrows indicate the direction of change of the differential equation for that particular combination of variable values. The red lines show where the differential equation is equal to zero, i.e. the nullcline of the equation. (A) Ai nullcline with respect to fbp concentration for all fbp concentrations greater than zero. We do not show the Ai nullcline with respect to pep concentration because the pep concentration does not affect the position of the Ai nullcline. (B) Pep nullclines with respect to external acetate concentration (at a fbp concentration of 0.1 $\mathrm{mmol\,g^{-1}}$) and (C) with respect to fbp concentration (at an acetate concentration of 5.5 mM). For (B) and (C), the Ai concentration is assumed to be at the equilibrium determined by the fbp concentration. (D) Fbp nullcline with respect to pep concentration.

system. To find these points, one can determine where in the variable space each differential equation is equal to zero (Fig 1). For each ODE, this is a line through variable space that is called the nullcline. Points where all nullclines of the ODE system intersect are equilibrium points. In order to identify these nullclines, it is easiest to start with the differential equation governing the concentration of the acetate incorporation enzyme ($\mathrm{d}Ai/\mathrm{d}t$), whose nullcline we will call the Ai nullcline for convenience. This differential equation can be factored into two parts; the growth rate $c \cdot J_{An}(pep, fbp)$ and the difference between the current protein concentration and the protein production rate $P_{Ai}(fbp) - Ai$. Consequently, the enzyme concentration Ai remains constant ($\mathrm{d}z/\mathrm{d}t = 0$) if (i) cells are not growing or (ii) the protein number per cell is equal to the production rate (Fig 1a). Because $J_{An}(pep, fbp) = 0$ if and only if $fbp = 0$, $\mathrm{d}Ai/\mathrm{d}t = 0$ either when $fbp = 0$ (in this case Ai can be any non-negative real number) or when $Ai = P_{Ai}(fbp)$. Now, when the external acetate concentration ac is greater than zero, all points where $pep = fbp = 0$ and $Ai > 0$ will not be equilibrium points, because in that case the reaction rate $J_{Ai}(Ai, ac)$ will be positive, acetate floods into the cell and consequently the intracellular concentrations pep and fbp will increase away from zero. This means that $pep = fbp = 0$, $Ai > 0$ are equilibrium points if and only if there is no acetate that can be taken up ($ac = 0$). Conversely, for all external acetate concentrations, $pep = fbp = Ai = 0$ is an equilibrium point. The reason is that without the acetate incorporation enzyme no acetate can be incorporated, and without intracellular metabolites no acetate incorporation enzyme can be produced.

An important point is that when $fbp > 0$ or $ac > 0$, $\mathrm{d}Ai/\mathrm{d}t$ is zero only if $Ai = P_{Ai}(fbp)$, and thus the Ai nullcline is entirely determined by the fbp concentration fbp (Fig 1a). Given that any equilibrium point must lie on the Ai nullcline, this also means that the equilibrium value $\hat{Ai}$ is determined by $\hat{fbp}$, and thus the deterministic system can be simplified at equilibrium by eliminating Ai. In other words, we can analyse the reduced system

$$\begin{aligned}\frac{\mathrm{d}pep}{\mathrm{d}t} &= \frac{1}{2} J_{Ai}(P_{Ai}(fbp), ac) - J_{Lg}(pep) - c \cdot J_{An}(pep, fbp) \cdot pep \\ \frac{\mathrm{d}fbp}{\mathrm{d}t} &= \frac{1}{2} J_{Lg}(pep) - J_{An}(pep, fbp) - c \cdot J_{An}(pep, fbp) \cdot fbp\end{aligned} \tag{13}$$

and proceed simply by identifying the nullclines of these two equations.

The nullcline of $\mathrm{d}pep/\mathrm{d}t$ (the pep nullcline) is determined by the rate of acetate incorporation $J_{Ai}(P_{Ai}(fbp), ac)$, which is determined by both the fbp concentration fbp and the external acetate concentration ac. Keeping fbp constant, the nullcline with respect to ac lies on a line determined by the Michaelis-Menten kinetics of the acetate uptake rate $J_{Ai}(P_{Ai}(fbp), ac)$ (Fig 1b). Conversely, when ac is constant, the pep nullcline is determined by the Hill kinetics of $P_{Ai}(fbp)$ (Fig 1c). As the external acetate concentration ac increases, the pep nullcline in pep-fbp space moves increasingly towards the right on the pep axis, i.e. to higher pep concentrations, until the cell reaches a maximum rate of Ai production, and the rate of acetate uptake saturates.

We next consider the fbp nullcline ($\mathrm{d}fbp/\mathrm{d}t = 0$). For each value of pep there is a unique value of fbp where fbp does not change. The converse is not true, however. That is, for each given value of fbp, there may be up to three concentrations of pep for which fbp does not change (Fig 1d). This asymmetry is a consequence of the MWC kinetics of $J_{An}(pep, fbp)$; at low pep, the rate of fbp production from pep $J_{Lg}(pep)$ increases faster with with greater pep concentrations than the rate of anabolism $J_{An}(pep, fbp)$. Therefore, the steady-state concentration fbp increases with pep. At a point determined by the dissociation constant of the enzyme An for its allosteric activator pep ($\mathrm{K}_{An,pep}$), the rate of fbp removal $J_{An}(pep, fbp)$ increases, which then lowers the steady-state fbp concentration. As the pep concentration increases further, An gets saturated with pep and the steady-state concentration of fbp starts rising again. The equation describing the change of fbp ($\mathrm{d}fbp/\mathrm{d}t$) thus plays an important role in introducing non-linearity into the system.

We next study equilibrium points of the two-equation ODE system (ODE system 13) by identifying intersections between the pep and the fbp nullclines. In particular, we will consider how the intersection points respond to changes in acetate concentration, because the pep nullcline moves along the pep axis in the pep-fbp space as ac changes. As the pep nullcline moves, the number of intersections between the fbp and the pep nullcline changes, which means that for a certain range of acetate concentrations there is a single equilibrium point, while for other acetate concentrations there may be two or three equilibrium points. This means that bistability exists only for a limited range of acetate concentrations.

At low values of the acetate concentration ac, the pep and fbp nullclines have a single intersection that lies at low pep and fbp concentrations (Fig 2a). As the acetate concentration increases, this intersection moves along the fbp nullcline towards both higher fbp and pep values until the fbp nullcline starts decreasing again on the fbp axis. In this region the pep nullcline intersects the fbp nullcline multiple times. With increasing ac and therefore higher steady-state pep concentrations, a second intersection appears at the lower end of this region. This second intersection splits, leaving one intersection travelling onwards down the fbp nullcline, and the other upwards towards lower pep and higher fbp concentrations (Fig 2b).

In this parameter regime there are thus three intersections, the two 'outer' intersections moving along the fbp nullcline towards higher values of pep, and one 'inner' intersection between them that is moving back towards lower pep and higher fbp values (Fig 2c). This latter intersection eventually joins with the original intersection still travelling down the fbp nullcline. The pep nullcline leaves the fbp nullcline at this point, leaving the intersection further down the fbp nullcline to continue onwards to higher pep concentrations (Fig 2d).

We can get some intuition about the stability of these intersections (which are equilibrium points) by visually inspecting how the ODE system behaves around these points (Fig 3a-b). If the ODE system moves towards an equilibrium point, that point is stable, while if it moves away from the point, the

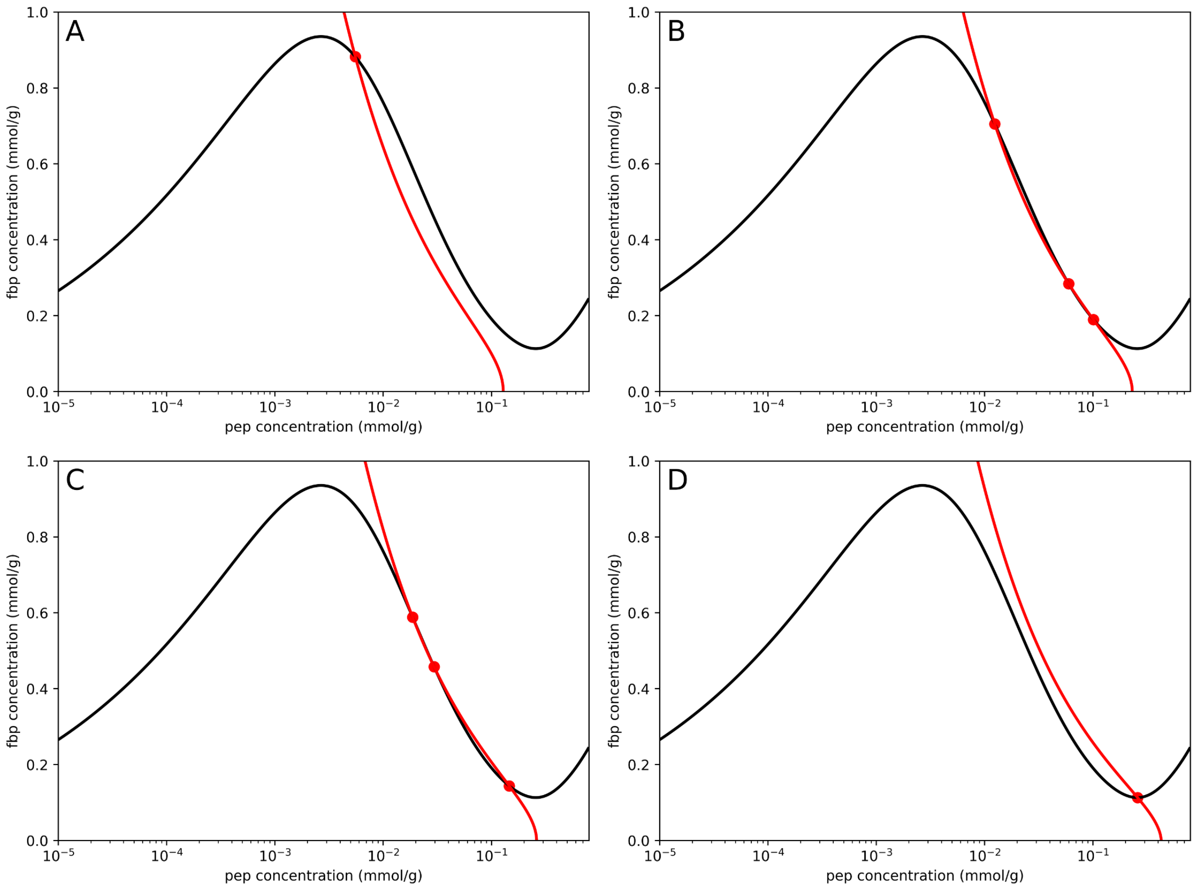

Figure 2: With increasing acetate concentration, the two-equation model (ODE system 13) goes through a region in which it has more than one equilibrium point. The pep and fbp nullclines are shown in red and black respectively, and the intersections between the two nullclines are marked by red circles. These intersections are equilibrium points. The panels show pep and fbp nullclines at (A) 3.0 mM acetate, (B) 5.4 mM acetate, (C) 6.1 mM acetate, and (D) 10.0 mM acetate.

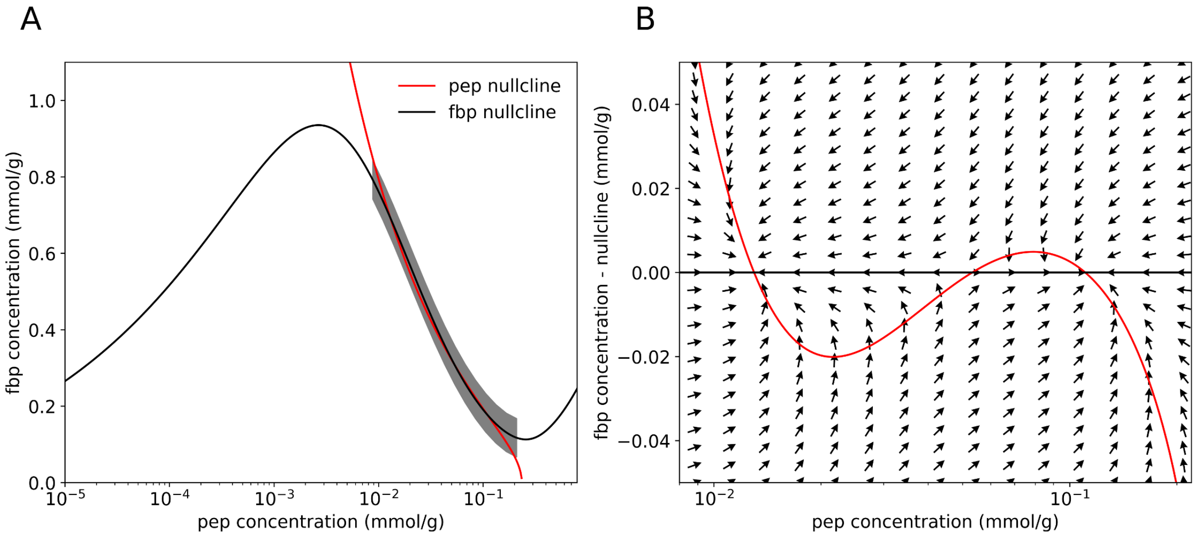

Figure 3: The two-equation ODE system can have up to three equilibrium points of which two are stable and one unstable. (A) The pep and fbp nullclines (shown in red and black respectively) have three intersections in the pep-fbp plane at an acetate concentration of 5.5 mM. Each intersection is an equilibrium point at which pep and fbp concentrations do not change. For better visibility, the grey shaded area is plotted again in (B), where the vertical axis is recast as the deviance in fbp concentration from the fbp nullcline. (B) The two outer equilibrium points are stable, and the point in between is unstable. The vector field shows the behaviour of the two-equation ODE system close to the three equilibria. Equilibria to which all arrows in the immediate neighbourhood point are equilibria to which the system converges. These equilibria are stable, because the system will return to these equilibria after perturbation. In contrast, if some arrows point away from an equilibrium (as is the case for the equilibrium in the middle), then the equilibrium is unstable and the system will diverge from it if perturbed.

equilibrium is unstable. If we examine the ODE system at an acetate concentration where there are three equilibrium points, we observe that the two outer points are stable and the inner point is unstable. Thus, at this acetate concentration the ODE system is bistable.

To determine the number and stability of equilibrium points for a range of acetate concentrations, we returned to the full ODE system with three differential equations. Using the python package sympy (version 1.1.1), we created expressions for the three nullclines and the Jacobian matrix of the full ODE system. Sympy was not able to find equilibrium points by solving the ODE system at a given acetate concentration. We thus used the expressions of the nullclines to find the equilibrium points numerically in sympy. To do so, we started with the observation that the fbp nullcline has only a single solution for each value of pep, but multiple solutions for some values of fbp. By choosing a given value for $\hat{pep}$, we could then solve for $\hat{fbp}$ numerically. We then obtained the value for $\hat{Ai}$ by inserting $\hat{fbp}$ and $\hat{pep}$ into the expression for the Ai nullcline ($\mathrm{d}Ai/\mathrm{d}t$) and solving for $\hat{Ai}$. Finally, we solved for the acetate concentration that would yield this equilibrium point by inserting $\hat{pep}$, $\hat{fbp}$, and $\hat{Ai}$ into the pep nullcline and solving for $\hat{ac}$. We then determined the stability of the equilibrium point by substituting the resulting values into the Jacobian matrix. Using this method, we investigated pep concentrations in the range of 10^{-10}-10^{2} $\mathrm{mmol\,g^{-1}}$. Towards the upper end of this range no equilibrium points exist because the production of the acetate incorporation enzyme Ai reaches a maximum beforehand. Therefore, the rate

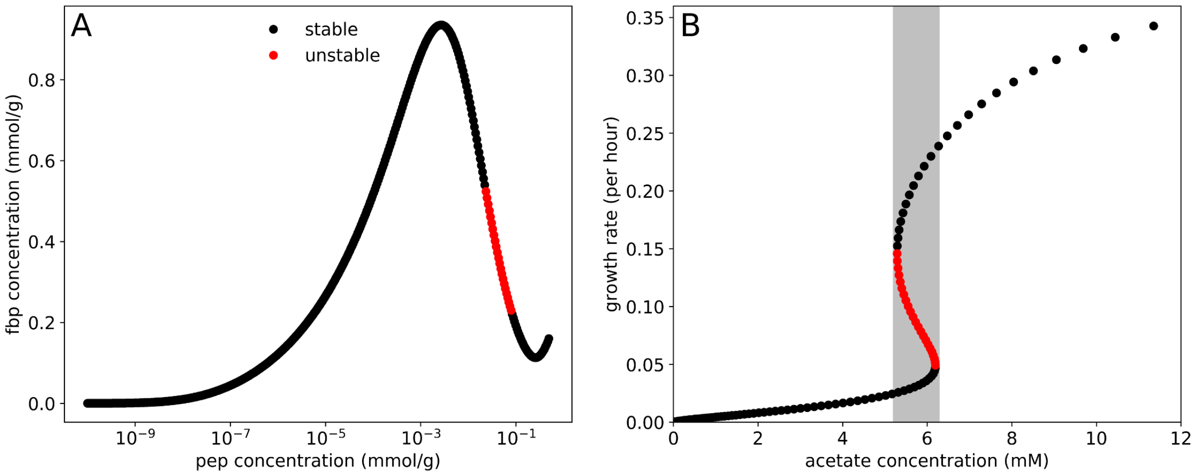

Figure 4: The system of three ODEs (ODE system 12) is bistable and shows hysteresis with regards to acetate concentrations. We numerically identified the equilibrium points and their stability over a range of pep concentrations (horizontal axis in A). Black and red circles show stable and unstable equilibrium points respectively. (A) The stable and unstable equilibrium points show two stable regions in the fbp-pep plane with an unstable region in between them. (B) The system shows hysteresis in growth rates to changes in acetate concentration. For some acetate concentrations (grey rectangle), two stable equilibrium points exist. In this region the ODE system shows hysteresis. That is, when acetate concentrations are slightly changed, the new equilibrium point depends on the system's previous acetate concentration: As acetate concentrations increase from a low value, the stable growth rate will jump to a high value at a relatively high acetate concentration (6.2 mM). Conversely, when the acetate concentration is lowered from a high value, the system will jump to a lower growth rate at a lower acetate concentration of 5.3 mM.

of pep production reaches a maximum, and consequently the *pep* nullcline does not advance further along the *pep* axis. The results confirmed the qualitative analysis above. In the pep-fbp plane (Fig 4a), two regions with stable equilibria are interrupted by a region containing unstable equilibria. This latter region occurs where the fbp nullcline decreases along the fbp axis while increasing along the *pep* axis. One of the points in this unstable region is the unstable equilibrium point described in the qualitative analysis. Taken together, these observations confirm our intuition that allosteric activation of the anabolic enzyme An is necessary to create bistability on acetate: If this allosteric activation of An by pep were not present, the fbp concentration would not decrease, and Cra activity would remain low, thus excluding growth on acetate.

When the equilibria are plotted in the plane described by the growth rate ($c \cdot J_{An}(pep, fbp)$) and the acetate concentration (Fig 4b) the system displays dynamics reminiscent of classic hysteresis. Nonlinear systems with hysteresis have memory effects: That is, the current system state depends on its previous state. This behaviour is apparent in the response of the growth rate to changing acetate concentrations. Because cells will avoid unstable growth rates (red circles in Fig 4b) the acetate concentration at which a cell jumps between a high and a low growth rate depends on whether the cell is currently experiencing slow or fast growth; the jump from fast to slow occurs at a lower acetate concentration than the jump from slow to fast growth. We note that when acetate concentrations change, the concentration of the acetate incorporation enzyme Ai in the full deterministic model with three ODEs does not travel along

Parameter kind	Parameters	Range	Reference
Metabolite dissociation constants	$K_{Cra,fbp}$	10^{-2} – 10^{6} mmol/g	[6]
	$K_{Ai,ac}$		
	$K_{Lg,pep}$		
	$K_{An,fbp}$		
	$K_{An,pep}$		
Promoter dissociation constant	$K_{Cra_A,DNA}$	10^{-1} – 10^{4} cell^{-1}	—
Turnover rates	$kcat_{Ai,s}$	10^{-1} – 10^{2} s^{-1}	[6]
	$kcat_{Lg,s}$		
	$kcat_{An,s}$		
Protein number	Cra	10^{-1} – 10^{4} cell^{-1}	[7]
	Lg		
	An		
Maximum protein number	Ai	10^{-1} – 10^{4} cell^{-1}	[7]
Hill coefficient	n_{Cra}	0 – 6	—
MWC cooperativity constant	n_{fbp}	0 – 9	—
MWC allosteric constant	L_{An}	10^{-7} – 10^{7}	—

Table 1: Parameters of the deterministic model (ODE system 12) and the range of their values used in sampling the parameter space. In the deterministic model, the number of all proteins except the acetate incorporation enzyme Ai are fixed.

the Ai nullcline determined by its production rate $P_{Ai}(fbp)$. Consequently, the trajectory of the three-equation ODE system in response to a change in extracellular acetate concentration will follow a more direct path than that given by the equilibrium points in the acetate-growth rate plane.

7 Searching parameter space: How likely is bistability, and what influences it?

In the previous section, we used a numerical analysis to confirm that a deterministic version of our model is bistable within a given range of acetate concentrations. However, on its own this observation might still mean that bistability of this kind is a rare and simply a peculiarity of our choice of parameter values. In other words, the bistability we simulate would not be robust to change in these parameters. If this were the case, then the mechanism we assume to cause bistability in carbon metabolism might also not persist over evolutionary time, because mutational changes might alter the biochemical parameters that allow bistability. We therefore searched the parameter space for combinations of parameters that lead to bistability in our model. The fraction of parameter samples that result in bistability is an estimate of the volume of parameter space that allows bistable behaviour, and is closely associated with its robustness to perturbations [5].

We used a simple, 'brute-force' approach to search the parameter space of our model. We created 48,092 samples from the 16-dimensional parameter space, where we drew parameter values at random from the ranges given in table 1. With the exception of dissociation constants, the MWC allosteric constant of the anabolic enzyme An (L_{An}), and the An MWC cooperativity constant (n_{fbp}), we sampled all parameters from a uniform distribution. For dissociation constants and the MWC allosteric constant

(L_{E4}) we sampled values as 10^x, where x was drawn from a uniform distribution. We did so because preliminary results indicated that low values preferentially led to bistability. The MWC allosteric constant L_{An}, is a ratio between the relaxed and tense forms of an enzyme [4] that determines the enzyme activity and the enzyme's response to a ligand that changes enzyme activity. Enzymes in their relaxed form are active, whereas enzymes in the tense form are inactive. Allosteric activators or inhibitors act by shifting the ratio between these two forms either towards the relaxed or the tense form respectively. The MWC allosteric constant is the ratio between these two states in the absence of the ligand, where a value of one means that both forms are equally likely [4]. We therefore sampled x from a uniform distribution in order to explore cases where either one or the other form predominates in absence of the An allosteric activator pep. For the MWC cooperativity constant n_{fbp} we sampled integers values, because the MWC cooperativity constant relates directly to the number of binding sites [4].

To determine if a parameter set leads to bistable behaviour, we used the same numerical approach as in the previous section, and again applied it to the deterministic model of three ODEs (ODE system 12). For each sampled parameter combination, we investigated 500 pep concentrations sampled at regular intervals on a logarithmic scale from 10^{-10} mmol g^{-1} to 10^{2} mmol g^{-1}. For each pep concentration, we then solved the system of ODEs for the corresponding fbp and Ai concentrations where the system lies at an equilibrium point (i.e. that all three ODEs are equal to zero). We then evaluated whether the equilibrium point thus identified was stable. Specifically, we used sympy to numerically determine whether the real parts of the eigenvalues of the Jacobian matrix at the equilibrium are negative. In this way, we obtained 500 equilibria along the pep concentration axis together with the stability of each equilibrium. If we found two regions with stable equilibria seperated by a region of unstable equilibria along the pep concentration axis (as in the previous section, Fig 4a), we concluded that the parameter set in question leads to bistable behaviour.

Overall, our sampling analysis showed that 1.5% of all parameter sets led to bistable behaviour. This percentage is probably an underestimate, as we examined only a modest range of pep concentrations. We observed that bistability can be found in a wide range of the parameter space we examined (Fig 5). For L_{An}, bistability becomes more likely as L_{An} increases because this means that allosteric activation by pep makes a greater impact on the activity of An (Fig 5g). A larger L_{An} means that in the absence of pep, the enzyme is less active. This result is consistent with a less formal analysis performed by Kotte et al. [8]. We also note that increased cooperativity of the anabolic enzyme An increases the probability of observing bistability (Fig 5l). A greater degree of cooperativity n_{fbp} makes the response of An to changes in the concentration of its activator pep more sigmoidal. Larger values of n_{fbp} make the response almost binary ('on-off'). This switch-like behaviour is conductive to bistability because it reinforces the difference between the growing (high rate of fbp removal, 'on') and nongrowing (low rate of fbp removal, 'off') state.

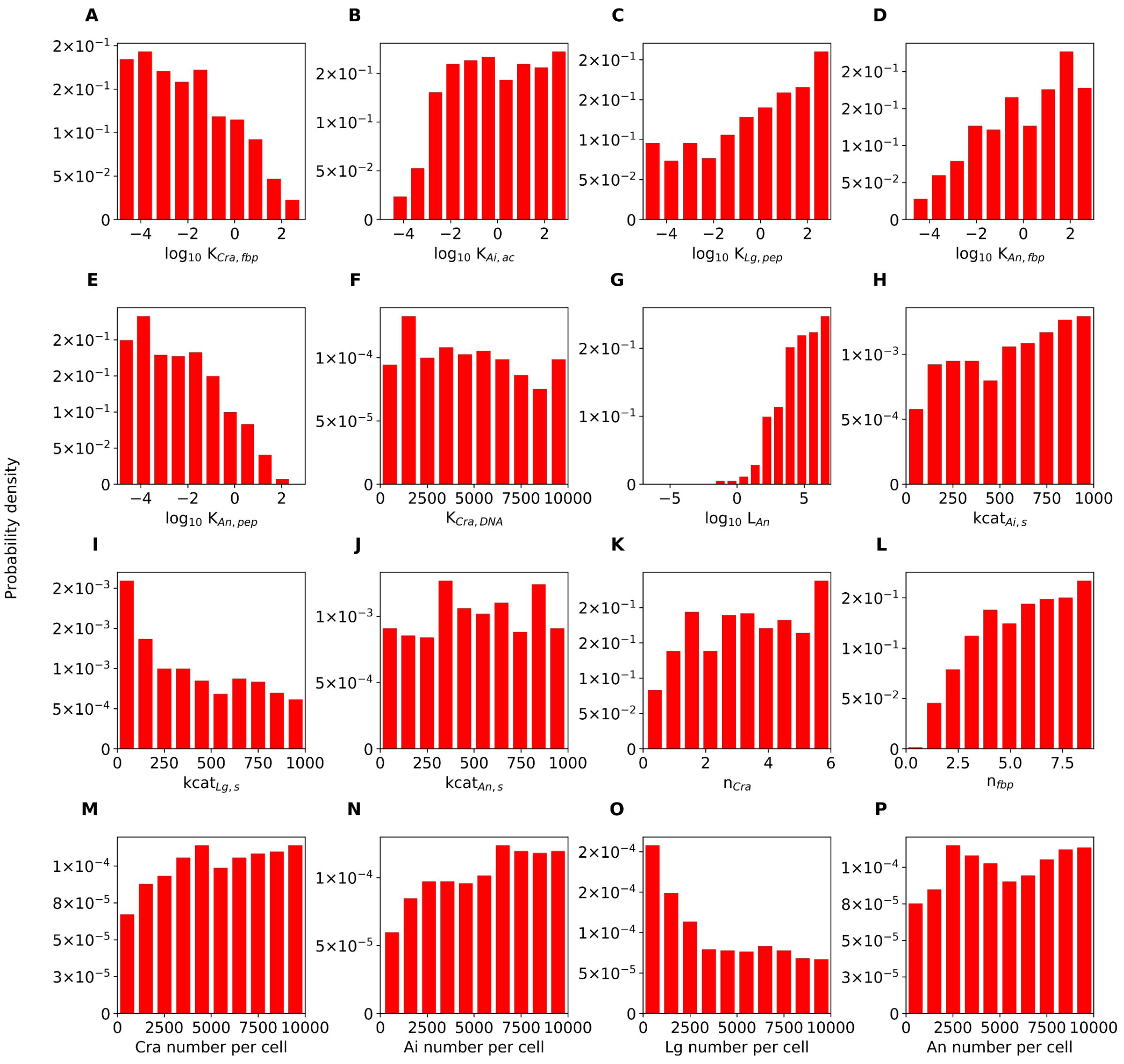

Figure 5: Distribution of the probability of finding bistable behaviour along each parameter axis. We generated 48,092 random parameter sets in a 16 dimensional parameter space and assessed numerically if the parameter set lead to bistability in the deterministic model consisting of three ODEs (ODE system 12). 732 parameter sets were bistable.

When we examined the presence or absence of skew in the probability of bistability, we observed that bistability is most likely in those regions of parameter space where both the growing and nongrowing state are stable and as distinct from each other as possible. These results are consistent with our analysis in the previous section and the intuitions laid out in the main text. To illustrate how parameter values can influence the stability of either one or both growth states, we will consider parameters affecting the activity of the three metabolic enzymes required for growth on acetate (Ai, Lg and An). For each of these enzymes, activity can be affected by enzyme concentration, by how tight the enzyme binds to its substrate, and by the binding of allosteric ligands [9].

For instance, bistability is more likely if the dissociation constant of the acetate incorporation enzyme Ai for acetate is high, i.e. if Ai-acetate binding is loose (Fig 5b). A higher dissociation constant means that, everything else being equal, a cell starts growing only at higher acetate concentrations. This insensitivity to low acetate concentrations stabilizes the nongrowing state. At the same time, bistability is more likely if the turnover number (Fig 5h) or the maximum expression level (Fig 5n) of Ai is higher. Both of these lead to a faster rate of acetate incorporation when acetate concentrations are high, and helps to stabilize the growing state.

Within a cell at steady-state, lowering the activity of an enzyme can increase the intracellular concentration of its substrate, and vice versa [9]. For example, in the enzyme Lg (Fig 5c), which converts pep into fbp, loose Lg-pep binding facilitates the accumulation of pep and thus the activation of the anabolic enzyme An. This process is required for the growing state to be stable once acetate flows into a cell. A low Lg activity can be also be achieved in other ways. For example, bistability also becomes more likely when the amount (Fig 5o) or turnover number (Fig 5i) of Lg is low.

Another example is the anabolic enzyme An (Fig 5d). Bistability is more likely when it has a lower affinity for fbp; lower affinity increases fbp concentrations and favours the nongrowing state. This low affinity is complemented, however, by a strong binding affinity of An for pep (Fig 5e), so that if pep is present, the rate of the reaction draining fbp from the cell increases, and cells enter the growing state.

We note that neither the amount (Fig 5p) nor the turnover number (Fig 5j) of An has a strong effect on the probability of observing bistability. This is because both affect the maximum rate of anabolism, while bistability is primarily caused by the difference in the reaction rate of anabolism between the growing and nongrowing state.

8 The number of Cra proteins affects the probability of growth on acetate

To make sure that the number of Cra proteins per cell affects the probability that a cell resides in the growing and non-growing state, we investigated the consequences of changing the number of Cra

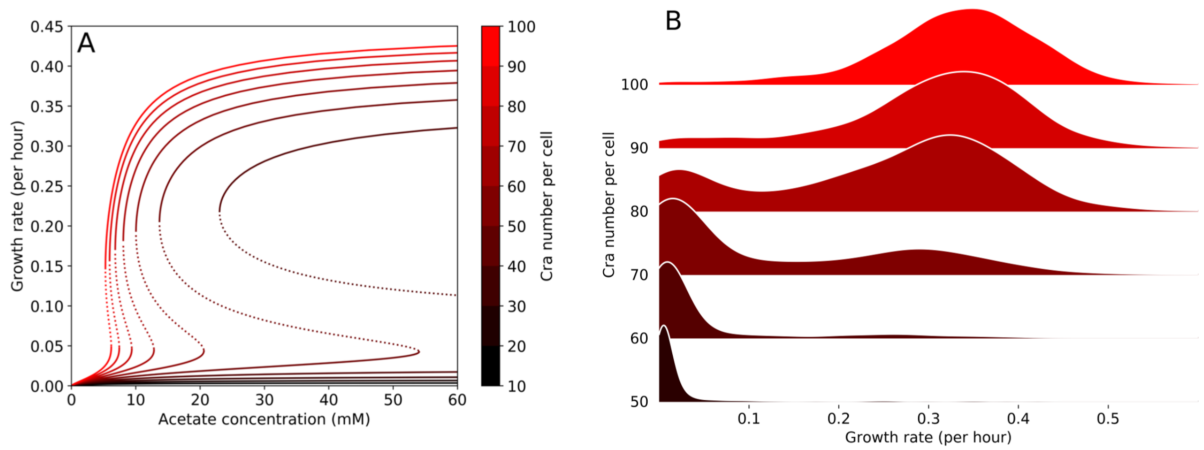

Figure 6: Larger number of Cra proteins per cell increases growth on acetate. (A) Equilibrium growth rates in the deterministic model consisting of three ODEs. As the number of Cra proteins per cell increases, cells shift from low to high growth rates at lower acetate concentrations. Solid lines show stable, and dotted lines unstable equilibria. (B) Gaussian kernel density approximation of the distribution of growth rates in the stochastic model, with a fixed number of Cra per unit biomass. As the number of Cra molecules increases, growth rates shift from slow to fast. Growth rates are shown for 1000 cells after 150 days of simulated growth in an unchanging environment containing 20mM acetate as the only carbon source.

molecules in (i) the 3-ODE deterministic model (equation 12) and (ii) our stochastic model, but with the amount of Cra fixed per unit biomass and cells growing in an environment that contains 20mM acetate at all times. In the deterministic model, increasing the Cra amount causes the steady-state growth rates of cells to shift from low to high at lower acetate concentrations (Fig 6a), and it increases the steady-state growth rate at all acetate concentrations. A similar effect is visible in the stochastic model (Fig 6b). Specifically, as the number of Cra molecules per unit biomass increases, the distribution of growth rates shifts from unimodal low growth rates (less than 0.1 h^{-1} with 50 Cra molecules per cell) to unimodal high growth rates (more than 0.2 h^{-1} with 100 Cra molecules per cell), with two modes clearly visible for Cra molecule numbers between 70 and 80 molecules.

9 Protein degradation does not remove the fitness benefit of noise when Cra-fbp binding is tight

Here we investigate the consequences of relaxing the assumption that proteins are not degraded. Proteins with a high turnover respond faster to changes in their production rate, and thus their concentrations are more sensitive to changes in gene expression [10]. Consequently, active degradation of the acetate incorporation protein Ai can make this protein more sensitive to variation in Cra activity, so that (i) cells may respond faster after the environment shifts to acetate and (ii) cell growth rate may become more vulnerable to random fluctuations in Cra numbers due to noise. Active protein degradation of Ai therefore has the potential to modify the fitness effects of Cra expression noise.

Active degradation of Ai may affect the circuit regulating a cell's response to an environment with

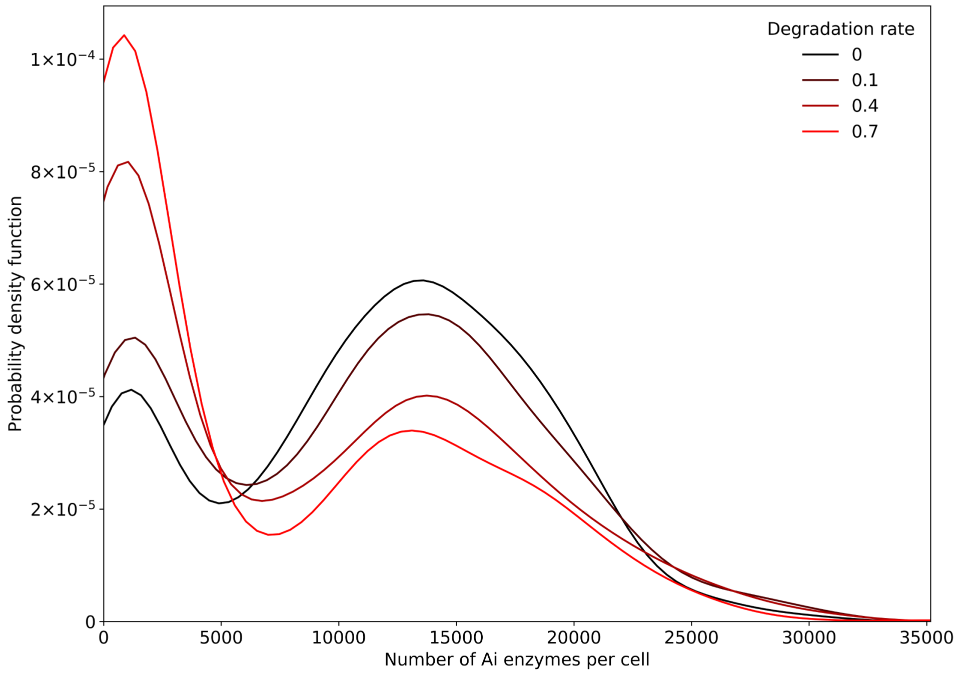

Figure 7: Active degradation of the acetate incorporation protein Ai shifts the population to a state in which Ai expression is low. Each line represents a Gaussian kernel approximation of the number of Ai proteins per cell in a population of 1000 cells. All cells in the population started with a high number of Ai (20,000 proteins per cell) and were left to equilibrate over 150 days in a constant environment with 20 mM acetate as the only carbon source. Upon cell division, only one of the offspring cells were kept. All cells had a Cra expression noise $\eta^2 = 0.2$.

only acetate, changing the probability at which cells occupy the growing and the nongrowing state. We simulated four degradation rates, with each 1000 cell lineages growing in an environment with a fixed concentration of acetate (Fig 7). We kept the average protein number per cell constant by simultaneously increasing the protein production rate k_1 (see Materials and Methods in main text). The distribution of the acetate incorporation protein Ai per cell shows two modes, with the high and low number of proteins corresponding to the growing and nongrowing state respectively. Increasing the degradation rate does not affect the positions of the two modes in the number of Ai proteins per cell. However, it does shift the population towards the nongrowing state. This decrease in the probability of growth occurs because fluctuations in Cra activity occur faster at high than at low growth rates, so that the growing state is more vulnerable to a random decrease in Cra activity than the nongrowing state is to a random increase.

We next considered how protein degradation may influence the bottleneck that populations experience after a switch to acetate. We chose a degradation rate at the upper end of the rates reported in *E. coli* (0.7 h^{-1}, BNID 109921, [11] and considered two Cra-fbp binding strengths (dissociation constants of 0.05 and 0.1 $\text{mmol}\,\text{g}^{-1}$, tighter and looser binding respectively). As in the main text, we modelled four levels of Cra expression noise. For each combination of Cra expression noise and the Cra-fbp dissociation constant, we simulated a population of cells growing for four days in an environment with constant nutrient flow. The environment switched from glucose to acetate after two days. We found that when

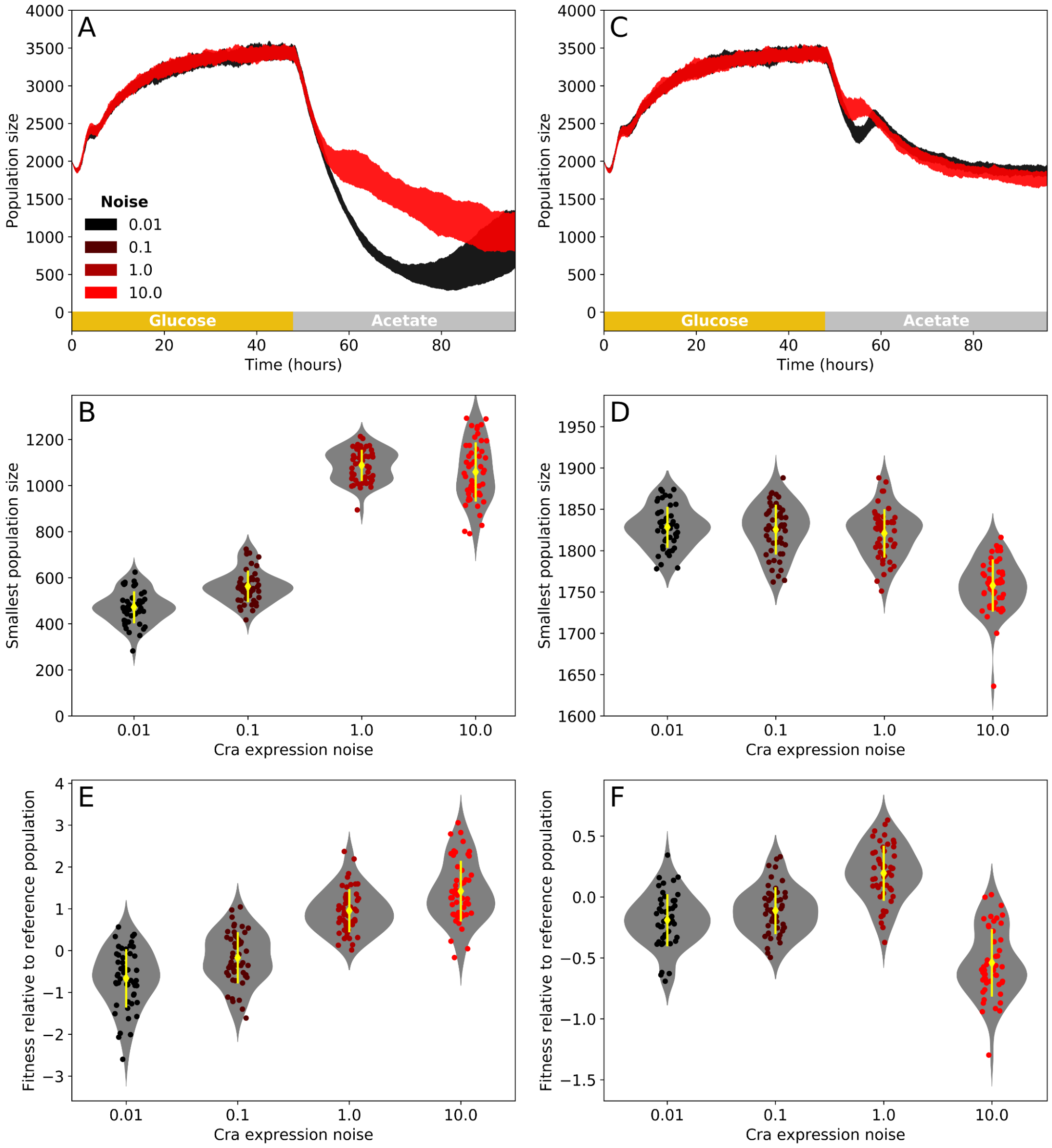

Figure 8: Active degradation of the acetate incorporation protein Ai leads to faster responses to a change in carbon source, but also causes lower steady-state population sizes on acetate. Consequently, the fitness effects of Cra expression noise switch from being beneficial at strong Cra-fbp binding to detrimental for the highest level of Cra expression noise at loose Cra-fbp binding. (A) Population size during two days of simulated growth in glucose (yellow box) and two days of growth in acetate (grey box) in a population with a Cra-fbp dissociation constant of 0.05 mmol g^{-1}. The areas in red and black show the range in population sizes for populations with the highest and lowest Cra expression noise respectively across 50 replicate simulations. (B) Smallest population sizes in acetate for populations with a Cra-fbp dissociation constant of 0.05 mmol g^{-1}. (C) Range of population sizes observed in 50 replicates for a population with a Cra-fbp dissociation constant of 0.1 mmol g^{-1}. (D) Smallest population sizes in acetate for populations with a Cra-fbp dissociation constant of 0.1 mmol g^{-1}. (E) Fitness of the population with a Cra-fbp dissociation constant of 0.05 mmol g^{-1} and (F) 0.1 mmol g^{-1} as determined through competition with a reference population with intermediate Cra expression noise, as well as with the same Cra-fbp dissociation constant and Ai degradation rate as the population that is being tested. In (B), (D), (E) and (F), yellow diamonds show the mean over 50 replicates and the yellow lines the standard deviation. Every circle shows the value observed in one replicate. The grey violin plots are Gaussian kernel approximations of the data. All populations actively degraded Ai at a rate of 0.7 h^{-1}.

Cra-fbp binding is tight (Fig 8a), noise in the expression of Cra makes a considerable difference in the trajectory of the population size. Instead of falling to a minimum and then recovering in population size, as happens in the two quieter populations (Cra expression noise $\eta^2 = 0.01$ and 0.1, smallest population size for $\eta^2 = 0.1$ is shown in Fig 8b), the populations with noisier Cra expression ($\eta^2 = 1.0$ and 10.0, the smallest population size for $\eta^2 = 10.0$ is shown in Fig 8b) decelerate their decline in population size much earlier, but continue falling until they reach the same cell number as the populations with quieter Cra expression (only shown for $\eta^2 = 10$ in Fig 8a). The result is that the two populations with higher Cra expression noise do not experience a bottleneck. The smallest population sizes in populations with low Cra expression noise are about 500 cells smaller than in populations with higher noise (Fig 8b). Differences in the smallest population size are statistically significant between populations with different levels of Cra expression noise (pairwise Wilcoxon rank sum test with Holm's correction, $p < 0.001$) except between the two noisiest populations ($p = 0.15$). In populations with looser Cra-fbp binding, population sizes do not fall beneath the carrying capacity in acetate at any level of noise (Fig 8c). Population decline temporarily stopped, and in the populations with the lowest Cra expression noise the population size even increases momentarily, before falling slowly towards the carrying capacity. As a result, the smallest population sizes are more indicative of the carrying capacity than a population bottleneck (Fig 8d). The mean smallest population size lies between 1820-1830 cells for all populations except for the noisiest (pairwise Wilcoxon rank sum test with Holm's correction, $p = 0.8$ for comparisons between all populations except with the noisiest population). In contrast, the noisiest population has a mean smallest population size of 1760 cells ($\pm$ 30 cells), which is significantly lower ($p < 0.001$ for all pairwise comparisons with the noisiest population). This decrease in carrying capacity (also visible in Fig 8c) is a consequence of the greater sensitivity to random fluctuations in Cra activity, which reduces the probability of maintaining growth on acetate (Fig 7). This increased sensitivity of Ai amounts to Cra activity also drives a reduction in the carrying capacity of populations with tight Cra-fbp binding (Fig 8a) compared to simulations without protein degradation (Fig 2a in the main text). Consequently, active degradation of the acetate incorporation enzyme is a double-edged sword: On the one hand, protein degradation increases the sensitivity of cells to the switch from glucose to acetate, and can cause populations to respond faster to elevated Cra activity. This response can be sufficiently fast to remove the bottleneck, as growing cells start dominating the population before the population is beneath the carrying capacity in acetate. On the other hand, protein degradation also decreases the carrying capacity on acetate.

We also investigated how protein degradation affects the relation between Cra expression noise and fitness at these two Cra-fbp binding strengths (Cra-fbp dissociation constants of 0.05 and 0.1 $\mathrm{mmol\,g^{-1}}$). We again estimated fitness through competition with a reference population, which also underwent degradation of the acetate incorporation enzyme. In populations with tight Cra-fbp binding (Cra dissociation

constant of 0.05 $\mathrm{mmol\,g^{-1}}$, Fig 8e), Cra expression noise increases fitness relative to the reference population (One-Way ANOVA, F=110.6, degrees of freedom 3 and 196, $p < 0.001$). All pairwise comparisons are significant (Tukey's HSD test, $p = 0.002$ for the comparison between the fitnesses of populations with Cra expression noise $\eta^2 = 10.0$ and 1.0, $p = 0.001$ for $\eta^2 = 0.1$ and 0.01, and $p < 0.001$ otherwise). In populations with loose Cra-fbp binding (Cra dissociation constant of 0.1 $\mathrm{mmol\,g^{-1}}$, Fig 8f), fitness differences are significant (One-Way ANOVA, F=85.0, degrees of freedom 3 and 196, $p < 0.001$). Fitness increases with Cra expression noise up to populations with the second highest level of Cra expression noise. However, populations with the highest Cra expression noise have on average the lowest fitness, even lower than that of the populations with the two quietest levels of Cra expression noise. The fitness values of the populations with the lowest level of noise are statistically indistinguishable (Tukey's HSD test, $p = 0.328$ for the pairwise comparison of the fitnesses of populations with Cra expression noise $\eta^2 = 0.01$ and 0.1, $p < 0.001$ for all others). The low fitness of the populations with the highest Cra expression noise occurs despite the fact that these populations resume growth the fastest (Fig 8a). However, this initial advantage is offset by the fact that these populations also have a lower carrying capacity (Fig 8d). Fitness differences between populations with different Cra expression noise are an order of magnitude lower than in populations with tighter Cra-fbp binding and protein degradation (Fig 8e) or in the absence of protein degradation (Fig 3a in the main text).

We therefore conclude that when Cra-fbp binding is sufficiently tight and Cra expression noise is low to intermediate, populations will undergo a bottleneck after a switch to acetate regardless of whether the acetate incorporation enzyme is actively degraded or not. In addition, increasing Cra expression noise results in a fitness benefit. Because nongenetic variation is predicted to be beneficial only in populations with low mean fitness, and tight Cra-fbp binding leads to low fitness (Fig 3b in the main text), we can explore the effects of Cra expression noise in the absence of active protein degradation without loss of generality.

10 Invasion probabilities of a neutral allele

To find out whether the impact of noise on the spread of a beneficial allele is caused by selection, we simulated the introduction of a neutral allele into a population. We also used these simulations to investigate how Cra expression noise influences the process of drift, because random nongenetic variation can affect the strength of drift [12, 13]. We simulated a population of 2000 cells, of which one carried a different allele at a neutral locus. We simulated growth in an environment that switches between glucose and acetate every two days, and continued the simulations until one of the two alleles had gone to fixation. The fixation of a neutral allele in an asexual haploid population occurs when the cell that carried that allele at the beginning of the simulation has become the ancestor of all surviving cells.

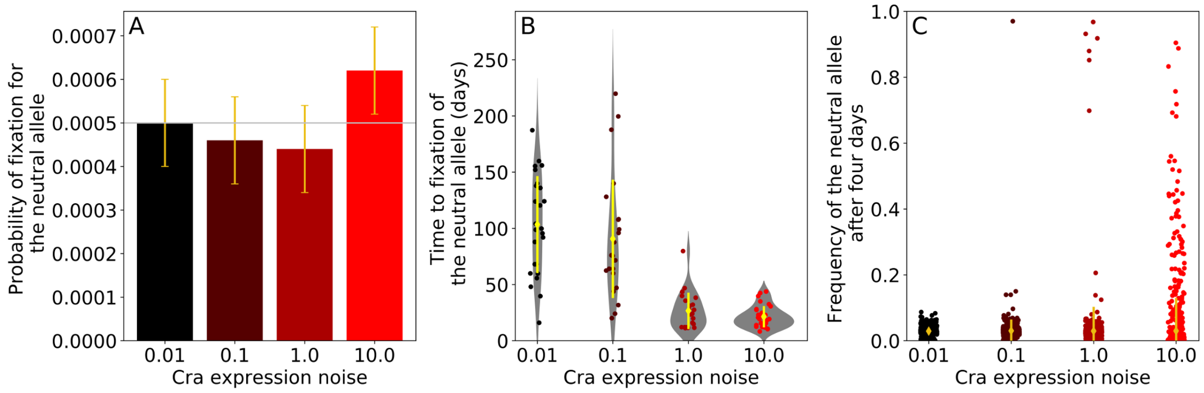

Figure 9: Gene expression noise does not influence the probability of fixation of a neutral allele, but does strengthen drift. (A) Probability of fixation of a neutral allele as observed in 50,000 replicate simulations each for four different levels of Cra expression noise. The probability of fixation is not significantly affected by noise (two-sided Fisher's exact test, p = 0.598, 50,000 replicates). Error bars show one standard deviation of the fixation probability under the assumption that the fixation probability follows a binomial distribution with a probability of success p = 0.0005, which is marked by the grey line. (B) Average fixation times decrease monotonically with Cra expression noise. From low to high Cra expression noise, the number of observations are 25, 23, 22, and 31. The grey violin plots are Gaussian kernel approximations of the data. (C) Frequency of the neutral allele after four days and one change in carbon source from glucose to acetate in simulations in which the allele has not been lost. Variation in the frequency of the neutral allele increases with Cra expression noise. From low to high Cra expression noise, the number of observations are 853, 843, 842, and 996. In (B) and (C), yellow diamonds show the mean and yellow lines indicate standard deviations. Every circle shows the value observed in one replicate.

Assuming that noise has no effect on the probability of fixation, such fixation occurs with a probability that is the inverse of the population size (N=2000) [14], and thus equals 0.0005. This probability of fixation is indeed close to the fraction of simulation runs in which the allele in question went to fixation (Fig 9a). The number of runs in which the neutral allele reaches fixation did not differ significantly between populations with different Cra expression noise (two-sided Fisher's exact test, $p = 0.598$, 50,000 replicates).

Greater nongenetic variation strengthens the influence of genetic drift on a population [12, 13], and therefore neutral alleles that do reach fixation should do so faster in populations with higher Cra expression noise. We observed that increasing Cra expression noise does indeed shorten fixation times. The average time to fixation of a neutral allele (Fig 9b) falls from 103 (± 43 standard deviation) days for the lowest Cra expression noise to 21 (± 4) days for the highest noise. We note that this acceleration occurs despite the fact that populations with high Cra expression noise experiences a less severe population bottleneck, which should give a rare neutral allele a better change to survive. Indeed, after four days of simulated growth, the number of replicate runs in which the neutral allele has not been eliminated is larger for the populations with high Cra expression noise (these are, from low to high noise, 853, 843, 842 and 996 simulation runs out of 50,000, Fisher's exact test, $p < 0.001$). Consequently, due to larger population sizes, the rare neutral allele has a higher change of surviving the first bottleneck. However, Cra expression noise increases the variation in the number of descendants that each cell in the initial population has after the first bottleneck, which increases the variance in the frequency of the neutral

allele after four days. The simulations with the highest Cra expression noise have the largest variance in the frequency of the neutral allele (Fig 9c). The standard deviations in allele frequency after four days are, from low to high noise, 0.004, 0.006, 0.010 and 0.015, an almost fourfold increase between the highest and the lowest level of noise. In contrast, the average allele frequency is close to 0.0005 (for the highest Cra expression noise, it is about 0.0006). The distribution of frequencies is significantly different between the simulations with the highest level of Cra expression noise and the other levels of noise, but not among the latter (Wilcoxon rank sum test with Holm's correction, $p = 0.009$ for pairwise comparison between $\eta^2 = 10$ and 0.01, $p = 0.005$ for comparisons between $\eta^2 = 10$ and 0.1 or 1.0, and $p = 1$ otherwise).

We therefore concluded that the differences in fixation probability of the beneficial allele that we observed in the main text are indeed due to its fitness difference to the wild-type allele. Also, we observed that while Cra expression noise does not affect the outcome of drift in terms of the fixation probability of a neutral allele, the fixation times are primarily driven by gene expression noise, and not by demographic stochasticity arising from small population sizes after the shift to acetate.

11 The benefits of Cra expression noise are less pronounced in environments that fluctuate randomly

Here we investigate whether Cra expression noise also results in faster fixation of a beneficial allele in environments that are fluctuating randomly rather than periodically. We simulated an environment that switches between glucose and acetate on average every 48 hours and a standard deviation of also 48 hours, with the duration of each period drawn at random from a gamma distribution. We used the same four levels of Cra expression noise (0.01 to 10) and the same alleles as in the main text, that is, a beneficial allele that loosens Cra-fbp binding (Cra-fbp dissociation constant of 0.07 $\text{mmol}\,\text{g}^{-1}$) and thus decreases lag times, and its competing wild-type allele (0.05$\text{mmol}\,\text{g}^{-1}$). Each simulation run started with an initial population size of 2000 cells and half of the population carrying the beneficial allele. The simulation continued until one of the competing alleles reached fixation.

Due to the increased environmental stochasticity, we observed a greater number of simulations where either the beneficial allele was lost and/or the population went extinct (Fig. 10a). In fact, because we allowed the simulation to continue until the population had finished the current glucose-acetate cycle, we observed several replicates in which one of the alleles reached fixation and the population subsequently went extinct. For example, in populations with the lowest Cra expression noise, four replicates lead to population extinction after the fixation of the beneficial allele. Consistent with our results in periodically fluctuating environments, the probability that the beneficial allele fixes in a population that survives until the end of the simulation increases with noise. Specifically, it increases from 86% to 100% from the least

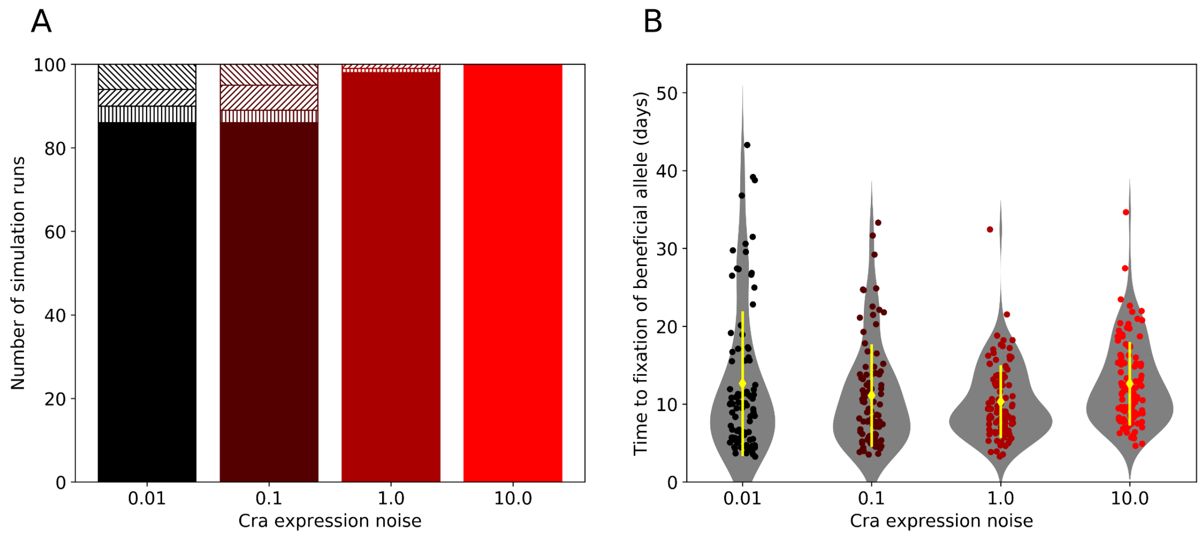

Figure 10: In randomly fluctuating environments, Cra expression noise increases the fixation probability of a beneficial allele but has a weak effect on fixation times when the allele does reach fixation. (A) The probability of fixation of a beneficial allele increases with Cra expression noise in an environment that fluctuates randomly between glucose and acetate. The duration of growth on either carbon source was determined by sampling from a gamma distribution with shape parameter 1 and scale parameter 48, leading to a mean switching time of 48 hours and a standard deviation of also 48 hours. The stacked bars show the number of simulations out of a total of a 100 replicates that resulted in one of four outcomes: The population survived until the end of the simulation and the beneficial allele became fixed (filled) or the wild-type allele became fixed (vertical strokes). Alternatively, the population went extinct, but first the beneficial allele reached fixation (right leaning strokes), or went extinct (left leaning strokes). (B) Cra expression noise has a weaker effect on fixation times of the beneficial allele than in periodically fluctuating environments. Yellow diamonds show the average time to fixation and yellow lines indicate one standard deviation. Circles show the fixation time of the beneficial allele in each replicate where the allele did reach fixation. Grey violin plots are Gaussian kernel estimates of the distribution of the data. Number of populations in which the beneficial allele reached fixation from lowest to highest Cra expression noise: 90, 92, 97 and 100.

noisy to the noisiest populations.

We also observed that the effect of Cra expression noise on the fixation times of the beneficial allele (if the allele reached fixation) is less pronounced than in environments that fluctuate periodically (Fig. 10b). The beneficial allele did reach fixation the fastest in populations that had the second highest level of Cra expression noise (10 days $\pm$ 5 days standard deviation), three days faster than in the populations with the highest (13 days $\pm$ 5 days) and lowest noise (13 days $\pm$ 9 days), and a day faster than the populations with the second-lowest level of noise (11 days $\pm$ 7 days). The differences in fixation time were still significant between the highest level of noise and the second and third highest levels of noise (pairwise Wilcoxon's rank sum test with Holm's correction, $p = 0.03$ for the pairwise comparison of $\eta^2 = 0.1$ with $\eta^2 = 10$ and $p = 0.007$ for the comparison between $\eta^2 = 1$ and $\eta^2 = 10$). In sum, expression noise is less able to accelerate the spread of a beneficial mutation in highly unpredictable environments.

References

[1] R Core Team. R: A Language and Environment for Statistical Computing. Vienna, Austria: R Foundation for Statistical Computing; 2015. Available from: https://www.R-project.org/.

[2] Hunter JD. Matplotlib: A 2D Graphics Environment. Computing in Science & Engineering. 2007;9(3):90–95. doi:10.1109/MCSE.2007.55.

[3] Labhsetwar P, Cole JA, Roberts E, Price ND, Luthey-Schulten ZA. Heterogeneity in protein expression induces metabolic variability in a modeled Escherichia coli population. PNAS. 2013;110(34):14006–14011. doi:10.1073/pnas.1222569110.

[4] Monod J, Wyman J, Changeux JP. On the nature of allosteric transitions: A plausible model. Journal of Molecular Biology. 1965;12(1):88–118. doi:10.1016/S0022-2836(65)80285-6.

[5] Wagner A. Circuit topology and the evolution of robustness in two-gene circadian oscillators. PNAS. 2005;102(33):11775–11780. doi:10.1073/pnas.0501094102.

[6] Bar-Even A, Noor E, Savir Y, Liebermeister W, Davidi D, Tawfik DS, et al. The Moderately Efficient Enzyme: Evolutionary and Physicochemical Trends Shaping Enzyme Parameters. Biochemistry. 2011;50(21):4402–4410. doi:10.1021/bi2002289.

[7] Taniguchi Y, Choi PJ, Li GW, Chen H, Babu M, Hearn J, et al. Quantifying E. coli Proteome and Transcriptome with Single-Molecule Sensitivity in Single Cells. Science. 2010;329(5991):533–538. doi:10.1126/science.1188308.

[8] Kotte O, Volkmer B, Radzikowski JL, Heinemann M. Phenotypic bistability in Escherichia coli's central carbon metabolism. Mol Syst Biol. 2014;10(7):736. doi:10.15252/msb.20135022.

[9] Kacser H, Burns JA. The Molecular Basis of Dominance. Genetics. 1981;97(3-4):639–666.

[10] Alon U. An Introduction to Systems Biology: Design Principles of Biological Circuits. CRC Press; 2006.

[11] Milo R, Jorgensen P, Moran U, Weber G, Springer M. BioNumbers—the database of key numbers in molecular and cell biology. Nucleic Acids Res. 2010;38(suppl 1):D750–D753. doi:10.1093/nar/gkp889.

[12] Mineta K, Matsumoto T, Osada N, Araki H. Population genetics of non-genetic traits: Evolutionary roles of stochasticity in gene expression. Gene. 2015;562(1):16–21. doi:10.1016/j.gene.2015.03.011.

[13] Wang Z, Zhang J. Impact of gene expression noise on organismal fitness and the efficacy of natural selection. PNAS. 2011;108(16):E67–E76. doi:10.1073/pnas.1100059108.

[14] Kimura M. On the Probability of Fixation of Mutant Genes in a Population. Genetics. 1962;47(6):713–719.
