# Supplementary material for: Gene expression noise can promote the fixation of beneficial mutations in fluctuating environments
Source: PLoS Comput Biol. 2020 Oct 26;16(10):e1007727. doi: 10.1371/journal.pcbi.1007727 (PMC7644098; doi:10.1371/journal.pcbi.1007727)
Supplement: Attachment — Submitted filename: Schmutzer and Wagner review.pdf [file pcbi.1007727.s002.pdf]

Major comment:
The authors have implemented a somewhat complicated individual based model to understand whether an idealised, contrived transcriptional circuit that was designed to exhibit bimodal growth behaviour can recapitulate what has been observed in bacterial populations known to exhibit bimodality.

I had a tough time with this paper. My major issue is perhaps that the authors seem to have been so preoccupied with whether or not they could, they didn't stop to think if they should [build the model]. In other words: yes - you can build the model. But are we actually learning anything from this model?

For example (lines 462-465):
**From a broader perspective*, our study highlights two especially important properties of population bottlenecks in fluctuating environments. The first is that the capacity to mitigate population bottlenecks can be an important part of adapting to fluctuating environments. The second is that a particular kind of bottleneck called a selective bottleneck can increase the rate of adaptation.*
I would argue that the first claim is well known - it is important to maximise the geometric mean of the population size during adaptation - it is well established that the geometric mean is what determines fitness over time. The second claim is well established (the authors cite Boag and Grant 1981 as having shown this and Moxon and Kussell 2017, who showed this *analytically*).

Additional comments:

1. Line 61 - 68: “Lag times can be caused by two different mechanisms.” This is a contrived dichotomy. There is good evidence that both of these play a role when carbon sources change and cell growth states are quantitatively monitored

2. Line 71: *Once the environment switches back to the superior carbon source glucose, all cells resume growth and no lag phase occurs at the population level.* This statement disregards the fact that cells exhibit memory for limited times, and if enough generations occur, cells “forget” the presence of the previous carbon sources, and *do* exhibit a lag phase (e.g. Lambert and Kussel 2014 PLOS Genetics).

3. Lines 203-205 *Populations with shorter lag times may have a fitness advantage compared to populations with longer lag times, because the former spend more time growing exponentially and thus leave more descendants.* Here and elsewhere, statements such as this serve to trivialise the results (or suggest that they are trivial). In other words, if the converse were true (populations with a shorter lag phase have lower fitness), then my conclusion would be that that the model was wrong, and not that a new dynamic was discovered. If this is not true, and there is a believable alternative hypothesis (“populations with short lag times have lower fitness”), then the authors should explain this and the reasoning behind it, and that this aspect is included in their model.

4. Line 217: similar to above *The fitness benefit of nongenetic variation in a trait often diminishes as populations approach the optimum value for that trait*. If the model had not shown this, the conclusion is likely that the model is wrong. This result provides little new information about growth and regulation dynamics other than the model re-creates what we know from biological data.

5. Similarly: *In general, the greater the fitness benefit of a beneficial allele, the less time it takes for the beneficial allele to go to fixation*. This is a well-known result from years of population genetics. Fixation time is proportional to $1/s$, with s being the selective benefit. This again brings to the fore that for many of these results, the implication is just that the model is not wrong.

6. *We found that when the initial frequency of the beneficial allele is low, it is most likely to go to fixation in populations with the highest Cra expression noise*. I find this to be the first interesting result of the manuscript. It is not trivially true (although upon some thought, it is readily apparent that the probability a new beneficial mutation is lost does relate to population size, and that population size and selective advantage are intertwined due to the construction of the model).

7. Line 197-200 *Take significantly longer to rebound* - here and many places elsewhere - the effect size is critical to note.

Line 53: *wherher* → whether
Lin 191: *quietest* → least noisy
