# Supplementary material for: Gene expression noise can promote the fixation of beneficial mutations in fluctuating environments
Source: PLoS Comput Biol. 2020 Oct 26;16(10):e1007727. doi: 10.1371/journal.pcbi.1007727 (PMC7644098; doi:10.1371/journal.pcbi.1007727)
Supplement: Attachment — Submitted filename: Schmutzer_Wagner_reply_to_reviewers.docx [file pcbi.1007727.s003.docx]

Dear Mr Schmutzer,

Thank you very much for submitting your manuscript "Gene expression noise can promote the fixation of beneficial mutations in fluctuating environments" for consideration at PLOS Computational Biology.

As with all papers reviewed by the journal, your manuscript was reviewed by members of the editorial board and by several independent reviewers. In light of the reviews (below this email), we would like to invite the resubmission of a significantly-revised version that takes into account the reviewers' comments.

We cannot make any decision about publication until we have seen the revised manuscript and your response to the reviewers' comments. Your revised manuscript is also likely to be sent to reviewers for further evaluation.

When you are ready to resubmit, please upload the following:

[1] A letter containing a detailed list of your responses to the review comments and a description of the changes you have made in the manuscript. Please note while forming your response, if your article is accepted, you may have the opportunity to make the peer review history publicly available. The record will include editor decision letters (with reviews) and your responses to reviewer comments. If eligible, we will contact you to opt in or out.

[2] Two versions of the revised manuscript: one with either highlights or tracked changes denoting where the text has been changed; the other a clean version (uploaded as the manuscript file).

Important additional instructions are given below your reviewer comments.

Please prepare and submit your revised manuscript within 60 days. If you anticipate any delay, please let us know the expected resubmission date by replying to this email. Please note that revised manuscripts received after the 60-day due date may require evaluation and peer review similar to newly submitted manuscripts.

Thank you again for your submission. We hope that our editorial process has been constructive so far, and we welcome your feedback at any time. Please don't hesitate to contact us if you have any questions or comments.

Sincerely,

Mark Goulian
Guest Editor
PLOS Computational Biology

Mark Alber
Deputy Editor
PLOS Computational Biology

***********************

Dear Dr. Goulian and Dr. Alber,

Please find enclosed a revised version of our manuscript. We would like to thank the three reviewers for their comments and suggestions, which helped us make the presentation of our results clearer and allowed us to define the scope of our results more precisely. In particular, we have taken into consideration randomly fluctuating environments, and find that Cra expression noise is less effective in accelerating the spread of beneficial mutations in such environments. We would also like to thank the editors for the opportunity to resubmit to PLOS Computational Biology.

Please find below a detailed reply to the reviewers.

With kind regards,

Michael Schmutzer and Andreas Wagner

Reviewer #1: This paper uses a mechanistic model of stochastic switching between glucose and acetate metabolism in a microbe to explore the effects of gene expression noise on both fitness and evolvability. I think this topic is ripe for modeling contributions of this sort and also perfect for the journal. The modeling approach and the evolutionary analysis both seem good, though there is some room for polishing of the presentation.

I was quite confused about the construction of beneficial alleles--these were introduced as "confer[ring] a fitness advantage that is independent of Cra expression noise", but then Fig. 4 demonstrates how this benefit is not independent of expression noise.

We thank the reviewer for pointing out this inaccuracy and ambiguity – our mistake. The fitness effects of this beneficial allele, which increases Cra-fbp affinity, are indeed *not* independent of Cra expression noise. What we meant was that the Cra-fbp affinity can be changed independently of the Cra expression noise. We changed this phrase to (page 8, lines 241-244) “In this section we will investigate whether Cra expression noise can accelerate the spread of a beneficial allele that also decreases lag times. This beneficial variant loosens Cra-fbp binding by increasing the Cra-fbp dissociation constant, thus increasing Cra activity.”

(Also, Fig. 4 could benefit from more replication; it's hard to get much out of panel B).

We apologise, panel 4B was hard to interpret because the smallest population sizes differ by an order of magnitude between treatments. To improve visibility, we have made the vertical axis logarithmic (see the left hand figure below). We have also run 50 additional replicates (for a total of a 100 replicates, see the right hand panel below) but as this did not greatly change the results we have kept this section as it is and report on the original set of 50 replicates.

It was hard to follow the set-up for this section--the hypothesis that noise must boost adaptation rate by boosting fitness effects seemed to come out of nowhere. The results that follow are interesting but somewhat hard to link with typical evolutionary theory results in simpler models. The benefits of noise for selection of a beneficial mutation seem to be a complicated blend of increasing geometric mean fitness (or just increasing fitness in the acetate environment without cost in glucose) and within-generation bet-hedging following a bottleneck. I would like to see this half of the paper clarified quite a bit, with a more clear set-up and better connection with the evolutionary literature on bet-hedging. This criticism is mitigated by the clear differentiation of these different effects in the Discussion; it is mostly in the Results that I would suggest revision.

The notion that noise increases the rate of adaptation by increasing the fitness consequences of a beneficial mutation originally comes from (Zhang, Qian & Zhang, 2009). The reviewer is correct in stating that fitness increases in acetate without incurring a fitness cost in glucose. There consequently is no bet-hedging in our model, but merely an increase in fitness on acetate. Our analysis in this section was also motivated by Moxon & Kussell (2017) work on selective bottlenecks, and we have rewritten the paragraph introducing this section in an effort to clarify this issue. The set-up to this section now reads (page 8, lines 239-253):

“In the previous two sections we have shown that Cra expression noise decreases the lag time, mitigates the severity of the population bottleneck in acetate (Fig 2a and b), and therefore increases fitness. In this section we will investigate whether Cra expression noise can accelerate the spread of a beneficial allele that also decreases lag times. This beneficial variant loosens Cra-fbp binding by increasing the Cra-fbp dissociation constant, thus increasing Cra activity.

Previous theoretical work has established that selective bottlenecks, i.e. bottlenecks in which two competing genotypes differ in their ability to survive a bottleneck, can accelerate adaptation [42]. The greater the relative advantage of a genotype in bottleneck survival, the faster the genotypes reaches fixation, especially when the bottleneck is severe. We therefore investigated whether Cra expression noise influences how effective the beneficial allele is at mitigating the population bottleneck in acetate. We then examined the effect of these population dynamics on the fitness of the beneficial allele before considering the rate at which the beneficial allele spreads through a population.”

Overall, I think this is a strong contribution that will interest readers in microbiology, systems biology, and evolution, and would be suitable for publication after a minor revision.

Notes (written as I read to highlight points of confusion)
Lines 15-26: There's a broader literature here that you might want to reference based on nongenetic phenotypic heterogeneity smoothing out fitness landscapes. I'd recommend Frank (2011) JEB and Whitlock (1997) Evolution.

We thank the reviewer for these two references, which turned out to be very useful, and which we now cite in the introduction (page 2, lines 15-23). In particular, we now draw on the concept of fitness smoothing from (Frank, 2011) to introduce the idea that nongenetic variation can benefit adaptation. We do so in the third paragraph of the introduction (page 2, lines 15-16): “On the other hand, nongenetic variation can increase the rate of adaptation by smoothing the fitness landscape [13].”

Lines 72-74: Is this really a prediction, or a premise of the way you designed the model?

The reviewer is correct – this is a premise of a model. For clarification, we changed the sentence in question to read (page 3, lines 69-73): “Empirical observations [24] suggest that once the environment switches back to the superior carbon source glucose, all cells resume growth and there is no division of the population into growing and nongrowing subpopulations. Our model incorporates these observations by assuming that cells growing on acetate do not have a growth disadvantage in glucose.”

Fig. 1B: Could you add a small plot of mean fop and pep concentrations for each bin of growth rate on acetate? It's not strictly necessary but might help reinforce the text.

We thank the reviewer for this suggestion. In response we have added an inset showing the mean concentrations to Fig. 1B.

Fig. 2A: This didn't render well for me; can't distinguish the four treatments. Recommend rethinking colors and approach here.

We apologize for the confusing figure design, we deliberately show only the trajectories of the noisiest and the least noisy populations in panel 2A. If we were to plot all four treatments, the trajectories would overlap and the figure would be hard to interpret. We now realize that placing the figure legend showing four treatments in panel 2A raises the expectation that all four treatments are plotted in that panel. We therefore moved the legend to panel C and added a sentence to the legend to panel 2A: “For clarity, we only show results from the noisiest and least noisy populations in this panel.”

Line 202: Could there be costs of noise during growth on glucose in your model? If so, I would prefer to measure both the costs and benefits by teasing them apart; if not, then it seems clear there will be a benefit of higher noise and I would change the focus to quantifying them, rather than reporting ANOVA results.

We did not incorporate any costs of noise on glucose in our model. We thank the reviewer for the suggestion. In response, we now added a quantification of the lag times on page 6, lines 197-202, which now read “The least noisy populations also take – on average – twice as long as the noisiest populations to rebound and reach carrying capacity (we use the term ‘carrying capacity’ in the sense of equilibrium population size), from 17 hours (± 1 hour) after the switch to acetate for the populations with the highest noise to 33 hours (± 2 hours) for the population with the lowest noise (Fig 2c).”

Lines 238-239: This is too narrow, as there are several distinct ways that expression noise could affect the rate of adaptation. Additionally, noise could complement mutations that affect any life-history trait, not just lag reduction--were more generic types of beneficial mutations considered? So, to me this is too strict in two dimensions. The section title also hints that you're going in a different direction--changing effective population size rather than the fitness benefit. Or, is this going to be about geometric mean fitness--but that's not what "ratio of bottleneck sizes" invokes for me. Overall, I found that the paper gets quite confusing in this paragraph.

We apologise about the confusing introduction to this section. We have rephrased this paragraph to clarify the motivation for our approach (page 8, lines 239-253, quoted above in our third reply). Briefly, in our model, population size and fitness are closely linked. We focus on bottlenecks in this section because previous work has shown that selective bottlenecks can accelerate adaptation (Moxon & Kussell, 2017). We aimed to show that Cra expression noise increases the selective advantage of a beneficial allele in a selective bottleneck, thereby increasing the rate at which the beneficial allele spreads. We refrained from discussing the fitness effects in terms of geometric fitness, because cells that do not grow on acetate decrease fitness in acetate without any benefit in glucose. Consequently, there is no bet-hedging in our model. While geometric fitness is necessary to explain why bet-hedging is advantageous, we believe that invoking geometric fitness to explain why noise improves fitness on acetate adds an unnecessary layer of complexity to our analysis.

We chose not to investigate the effect of Cra expression noise on other kinds of mutation, because the paper is already long and complex. Each additional mutation would have required adding three figures analogous to Fig. 4. In addition, each mutant might behave differently, such that a thorough discussion would have rendered the paper even harder to read. We hope that the reviewer will understand.

Reviewer #2: Using agent based simulations, Schmutzer & Wagner investigate the effect of noise in Cra expression on the evolutionary dynamics and fitness of a population of E.coli that undergoes a change from exponential growth on glucose to growth on acetate. They show that the presence of noise in the repressed state of Cra (under glucose conditions) is beneficial for bacteria when they switch to acetate, as this leads to higher fitness of the population and a decrease in bottlenecks due to the fact that Cra is repressed in the initial condition.

My main points of criticism are:

1. The study considers only switching from one condition to the other (glucose to acetate) after being in very long term exponential growth, i.e. chemostat conditions. Such conditions are really the exception in nature, as most microbes live under repeated feast and famine conditions, namely cells in stationary phase will encounter a new type of nutrient. Hence one should be rather careful in generalizing the results obtained from such idealized initial setup.

Great point. Yes feast and famine scenarios are likely widespread in nature. That our model is not suitable for investigating feast and famine scenarios is a serious limitation of the model, which we now mention explicitly in the discussion (page 12, lines 381-384): “Many bacteria regularly encounter feast and famine conditions [43,44], and lag times commonly occur after long periods of starvation. The underlying regulation and physiology of these lag times are distinct from the case we have simulated here [43,45].” We revisit this issue at the end of the discussion (page 14, lines 492-496): “Most non-pathogenic organisms also experience selective bottlenecks, because natural environments are heterogeneous and unpredictable [45, 62, 64, 65]. While such fluctuations are often random or involve feast and famine scenarios, others are periodic. Examples include the intensity of predation of lynxes on hares [66] and seasonal changes in food availability [67] or temperature [68].”

2. The authors consider switching events between the two food sources in fluctuating environments that are periodic, again not very typical in nature. In order to have a more general view of the effect of noise on fitness in this specific system, especially given the fact that Cra it is a global regulator, it would be helpful to explore how the populations fare in non-periodic fluctuating environments, namely with variable switching times between environments. Given that the results are simulations based and do not rely on analytical solutions this I find crucial.

We thank the reviewer for this suggestion. In response, we have performed additional simulation in stochastically varying environments, and discuss the results in the new supplementary materials section 11. In environments that fluctuate randomly, Cra expression noise still protects the beneficial allele from extinction, but is less able to accelerate the allele’s spread through the population. We refer to this new analysis in the manuscript:

In the results (page 11, lines 350-352): “Both of these effects can also be observed, albeit to a lesser extent, in environments that fluctuate randomly instead of periodically between glucose and acetate (see S1 Appendix section 11).”

In the discussion (page 12, lines 418-421): “However, we do caution that this effect is most pronounced in environments that fluctuate periodically. When environments fluctuate randomly, this effect is attenuated (S1 Appendix section 11).” And (page 13, 425-427): “This effect also increases the eventual fixation probability of the beneficial allele and is robust in environments that fluctuate randomly.”

3. The numbers of Cra molecules used for the stochastic part of the model (lines 149 to 155 or so) do not seem to come from experimental determinations of Cra molecules in the cell. A mean of 100 molecules seems low for a global regulator.

The reviewer is entirely correct in stating that 100 molecules is low for a global regulator, but under the slow growth conditions we study, existing evidence suggests that this number may be of the right order of magnitude. We originally determined the mean number of Cra molecules from the model of Kotte et al (2010), so that the concentration of Cra molecules per unit mass would be of the same order of magnitude as in their model. Estimates from empirical studies depend on the growth rate considered. At one extreme, a recent study (Ishihama *et al.*, 2014) reports a Cra copy number of about 1000 molecules per genome in cells that are growing exponentially on a rich medium. In contrast, the main proteomics study we consulted (Taniguchi *et al.*, 2010, table S6, row 20) reported a mean of only 29 Cra molecules per cell. Its much lower estimate may reflect the fact that it comes from a slowly growing population (0.27 hr^-1^), a treatment that probably lowers the measured protein abundances compared to other studies (see figure S2D in Li *et al.*, 2014). We note that we also model slowly growing populations (0.1 hr^-1^). Because the issue is important, we now briefly discuss it in the description of our model (page 5, lines 158-159): “This concentration is low for a global regulator [38], but consistent with the slow growth conditions we simulate here [37,39].”

In addition, we note that this assumption is not likely to affect our results dramatically, because our sensitivity analysis (section 7 in the supplementary materials) shows that the mean number of Cra molecules per cell has only a small effect on the probability of observing bistability (figure S5M). In other words our model is robust to the average Cra concentration.

Thus, the generality claims need to be brought down a notch. I think we need to be a bit more careful with what noise does or does not do for biology and evolution, as we risk to fall into the trap of the Spandrels of San Marco again, by ignoring the wider context of how organisms evolve, in this case the feast and famine aspect and truly fluctuating nature of this dynamics as opposed to cyclic scenarios.

Point well taken. We have toned down claims of generality and now specify that our results mainly hold in periodically fluctuating environments. We have added corresponding caveats to the abstract and the discussion, and the conclusion.

In the abstract, last two sentences: “Central to the adaptation-enhancing principle we identify is the ability of noise to mitigate population bottlenecks, particularly in environments that fluctuate periodically. Because such bottlenecks are frequent in fluctuating environments, and because periodically fluctuating environments themselves are common, this principle may apply to a broad range of environments and organisms.”

In the discussion (page 12, lines 418-421): “However, we do caution that this effect is most pronounced in environments that fluctuate periodically. When environments fluctuate randomly, this effect is attenuated (S1 Appendix section 11).”

In the conclusion (page 14, lines 500-503): “We identify how noise can on the one hand accelerate the spread of a beneficial allele by enhancing its fitness benefit in a selective bottleneck that occurs periodically, and on the other hand protect the beneficial allele from extinction during the bottleneck.”

A few more minor things:
The text in general can be cut down as many things can be explained in a more brief manner. It is quite tedious to read in places.

We have edited the results to be more concise. Please note that in reply to this comment, we will refer to page and line numbers from the document with tracked changes. Otherwise, all line numbers refer to the new manuscript.

We removed this sentence (page 7, lines 211-212): “To find out whether Cra expression noise does increase fitness, we first studied how Cra expression noise influences the lag time after a shift from glucose to acetate.”

Page 7, lines 238-241: We replaced “Populations with shorter lag times may have a fitness advantage compared to populations with longer lag times, because the former spend more time growing exponentially and thus leave more descendants. To find out whether fitness does increase…” with “To quantify how much fitness increases…”

Page 8, lines 248-253: We replaced “…i. e. a one-way ANOVA reveals significant differences in fitness…” with “(one-way ANOVA, …)”

Page 8, lines 257-263: We replaced “The fitness benefit of nongenetic variation in a trait often diminishes as populations approach the optimum value for that trait [16,17,20]. We investigated whether this reduction also occurs in our model. Specifically, we hypothesised that the fitness benefit derived from the presence of cells with exceptionally high Cra activity may become irrelevant as a population’s lag time shortens, which is a proxy for its closeness to a fitness optimum. To test this hypothesis…” with “We also quantified how Cra expression noise changes the fitness landscape. To create this fitness landscape…”

Page 11, lines 353-356: We removed “In populations with high Cra expression noise (Fig 5a) the frequency of the beneficial allele increases consistently over time. In contrast, in populations with low Cra expression noise(Fig 5b), the beneficial allele also sometimes decreases in frequency…”

Page 11, lines 359-360: We removed “In general, the greater the fitness benefit of a beneficial allele, the less time it takes for the beneficial allele to go to fixation (Fig 5c).”

Page 11, line 371: We replaced “These differences in fixation time are…” with “The fixation time is…”

The use of yellow in several of the figures makes it hard to read the figures.

For the figures where we show yellow on white, we now use a darker colour. For violin plots, we believe the contrast between the bright yellow on the grey violin plot is sufficiently high, but we have thickened the error bars to improve visibility.

Reviewer 3

Major comment: The authors have implemented a somewhat complicated individual based model to understand whether an idealised, contrived transcriptional circuit that was designed to exhibit bimodal growth behaviour can recapitulate what has been observed in bacterial populations known to exhibit bimodality.

We respectfully disagree with the reviewer’s characterization of the aim of our paper. Our main quest in this paper was to answer the question “does gene expression noise affect the rate of adaptation in a fluctuating environment, and if so, how?” We did so by taking a systems-biology approach (Alon, 2006) that consists of building a model explicitly linking events that occur at the molecular scale to population dynamics and fitness in a way that would not be possible in a more phenomenological model. We took care to ensure that our model simulates the kind of bimodality seen in real-life bacteria (Kotte *et al.*, 2014), but we then go on to answer an evolutionary question that has not yet been addressed.

I had a tough time with this paper. My major issue is perhaps that the authors seem to have been so preoccupied with whether or not they could, they didn’t stop to think if they should [build the model]. In other words: yes - you can build the model. But are we actually learning anything from this model?

We take the view that our paper addresses a gap in the literature on gene expression noise and the rate of adaptation. Although it is well known that noise can increase fitness in fluctuating environments (for example Schreiber *et al.*, 2016), and that there can be a link between noise increasing fitness and noise increasing the rate of adaptation (Zhang, Qian & Zhang, 2009), before our study it was unknown whether this also holds in fluctuating environments and what mechanism would constitute this link. In fact, a recent preprint (Rocabert *et al.*, 2020) has shown that noise does not necessarily increase the rate of adaptation in environments where it increases fitness, reinforcing the need to examine what effects noise has on the rate of adaptation in fluctuating environments. We now explicitly make this last point in the introduction (page 3, lines 50-52): “In addition, a recent theoretical study has shown that even when nongenetic variation increases the mean fitness of a population, this does not necessarily lead to an acceleration in the spread of beneficial alleles [15].”

So yes, we do learn something new from this model: i) Gene expression noise can increase the rate of adaptation in fluctuating environments, and ii) the “missing link” between the fitness benefit of noise and the rate of adaptation in such environments are selective bottlenecks. We have emphasised in the discussion that these are two most important implications of our study (page 12, lines 403-404): “The most important finding of our paper is that Cra expression noise influences the spread and eventual fixation of this beneficial allele in two ways.”

For example (lines 462-465):

**From a broader perspective*, our study highlights two especially important properties of population bottlenecks in fluctuating environments. The first is that the capacity to mitigate population bottlenecks can be an important part of adapting to fluctuating environments. The second is that a particular kind of bottleneck called a selective bottleneck can increase the rate of adaptation.*

I would argue that the first claim is well known - it is important to maximise the geometric mean of the population size during adaptation - it is well established that the geometric mean is what determines fitness over time. The second claim is well established (the authors cite Boag and Grant 1981 as having shown this and Moxon and Kussell 2017, who showed this *analytically*).

Moxon and Kussel (2017) indeed play a key role in interpreting our results. Their model shows how selective bottleneck accelerates adaptation. However, what they do not show is how gene expression noise (which is one of the mechanisms, next to high mutation rates or epigenetic changes, by which the switching between alternative phenotypic states in their model might occur) affects selective bottlenecks and therefore the rate of adaptation. In their analytic model and in their stochastic simulations, the switching rate is independent of the bottleneck size, which gives their model added generality and reflects the fact that organisms often have little control over the factors that affect the severity of a bottleneck. Yet this latter assumption may not always hold, and here we have simulated an instance where the organism has some degree of control over the severity of a bottleneck. On a different note, we did not wish to make the claim in this paragraph that we have discovered these two principles, but aim merely to place our study into the context of the existing literature. We have added to the sentence in question that these two properties are well known. The sentence now reads (page 14, lines 477-478): “From a broader perspective, our study highlights two especially important and well known properties of population bottlenecks in fluctuating environments.”

Additional comments:

1. Line 61 - 68: “Lag times can be caused by two different mechanisms.” This is a contrived  dichotomy. There is good evidence that both of these play a role when carbon sources change and cell growth states are quantitatively monitored

We thank the reviewer for flagging this point; these causes are indeed not mutually exclusive. We have changed this sentence to (page 3, lines 60-63) “Lag times can have two (nonexclusive) causes [24]. The first, which affects all cells to a similar extent, is that cells need time to reconfigure their metabolism when the environment changes. The second is that only some cells may start to grow once a new carbon source has become available.”

2. Line 71: *Once the environment switches back to the superior carbon source glucose, all cells resume growth and no lag phase occurs at the population level.* This statement disregards the fact that cells exhibit memory for limited times, and if enough generations occur, cells “forget” the presence of the previous carbon sources, and *do* exhibit a lag phase (e.g. Lambert and Kussel 2014 PLOS Genetics).

This sentence refers to the observation of Kotte *et al* (2014) that *E. coli* populations do not exhibit bimodality after a shift away from a gluconeogenic carbon source to glucose (see their table S1). For clarification, we have changed this sentence to read (page 3, lines 69-72) “Empirical observations [23] suggest that once the environment switches back to the superior carbon source glucose, all cells resume growth and there is no division of the population into growing and nongrowing subpopulations.”

3. Lines 203-205 *Populations with shorter lag times may have a fitness advantage compared to populations with longer lag times, because the former spend more time growing exponentially and thus leave more descendants.* Here and elsewhere, statements such as this serve to trivialise the results (or suggest that they are trivial). In other words, if the converse were true (populations with a shorter lag phase have lower fitness), then my conclusion would be that that the model was wrong, and not that a new dynamic was discovered. If this is not true, and there is a believable alternative hypothesis (“populations with short lag times have lower fitness”), then the authors should explain this and the reasoning behind it, and that this aspect is included in their model.

We thank the reviewer for flagging this statement, and also the other problematic statements in points 4 and 5 below. This is bad writing on our part. We have replaced or eliminated these sentences. In response to point 3, we have changed the sentence to (page 6, lines 207-208): “To quantify how much fitness increases with shorter lag times, we simulated a competitive fitness assay.”

That there are situations in which short lag times are disadvantageous is well known (Moreno-Gámez *et al.*, 2020). However, we have not simulated such a case because it fell outside our main aim, and the paper is already long and complex. This is a limitation that we acknowledge in the discussion (page 12, lines 378-379): “We caution that our results are valid only for cases where selection favours decreasing, not increasing lag time.”

4. Line 217: similar to above *The fitness benefit of nongenetic variation in a trait often diminishes as populations approach the optimum value for that trait*. If the model had not shown this, the conclusion is likely that the model is wrong. This result provides little new information about growth and regulation dynamics other than the model re-creates what we know from biological data.

We have replaced this statement with (page 7, line 222): “We also quantified how Cra expression noise changes the fitness landscape.”

5. Similarly: *In general, the greater the fitness benefit of a beneficial allele, the less time it takes for the beneficial allele to go to fixation*. This is a well-known result from years of population genetics. Fixation time is proportional to 1/*s*, with *s* being the selective benefit. This again brings to the fore that for many of these results, the implication is just that the model is not wrong.

We have removed this sentence, we agree it is unnecessary.

6. *We found that when the initial frequency of the beneficial allele is low, it is most likely to go to fixation in populations with the highest Cra expression noise.* I find this to be the first interesting result of the manuscript. It is not trivially true (although upon some thought, it is readily apparent that the probability a new beneficial mutation is lost does relate to population size, and that population size and selective advantage are intertwined due to the construction of the model).

We thank the reviewer for pointing this out, and now mention this result more prominently in the abstract and the conclusion.

Lines 15-17 of the abstract now read: “Additionally, noise can protect this allele from extinction, accelerate its spread, and increase its likelihood of going to fixation.”

In the conclusion, we now state (page 16, lines 500-503): “We identify how noise can on the one hand accelerate the spread of a beneficial allele by enhancing its fitness benefit in a selective bottleneck that occurs periodically, and on the other hand protect the beneficial allele from extinction during the bottleneck.”

7. Line 197-200 *Take significantly longer to rebound* - here and many places elsewhere - the effect size is critical to note.

We have added the effect sizes at multiple points in the results.

Page 6, lines 197-202: “The least noisy populations also take – on average – twice as long as the noisiest populations to rebound and reach carrying capacity (we use the term ‘carrying capacity’ in the sense of equilibrium population size), from 17 hours (± 1 hour) after the switch to acetate for the populations with the highest noise to 33 hours (± 2 hours) for the population with the lowest noise (Fig 2c).”

Pages 6-7, lines 214-218: “Compared to the reference population, the least noisy population is on average slightly less fit than the reference population (relative fitness of -0.018 ± 0.085, zero indicates no fitness difference), while the noisiest population is substantially fitter (0.177 ± 0.056). These fitness differences between populations with different levels of noise are significant…”

Page 9, lines 277-282: “The average fitness benefit (Fig 4c) increases with noise from 1.13 (± 1.80) up to 3.53 (± 1.7) for a Cra expression noise of η^2^ = 1 (ANOVA, F=18.9, degrees of freedom 3 and 196, p < 0.001). For populations with the largest Cra expression noise (η^2^ = 10), we found a lower fitness gain (2.65 ± 0.99) from the beneficial allele than for populations with the second largest level of noise…”

Page 10, lines 303-304: “…with the fixation time falling from an average of 12 days (± 5 days) at the lowest noise to 9 days (± 2 days) for the second highest level of noise η^2^= 1…”

Page 10, lines 307-309: “The beneficial allele spreads considerably slower in populations with the highest Cra expression noise (11 days ± 2 days)…”

Page 10, lines 327-330: “We found that when the initial frequency of the beneficial allele is low, it is most likely to go to fixation in populations with the highest Cra expression noise, with the observed probability of fixation increasing from 0.003 to 0.013 in the least noisy to the noisiest populations…”

Line 53: *wherher* → whether Line 191: *quietest* → least noisy

We thank the reviewer for their keen eyes – corrected.

References

Alon, U. (2006) *An Introduction to Systems Biology: Design Principles of Biological Circuits*. CRC Press.

Frank, S.A. (2011) Natural selection. II. Developmental variability and evolutionary rate*. *Journal of Evolutionary Biology*. 24 (11), 2310–2320.

Ishihama, A., Kori, A., Koshio, E., Yamada, K., et al. (2014) Intracellular Concentrations of 65 Species of Transcription Factors with Known Regulatory Functions in Escherichia coli. *Journal of Bacteriology*. 196 (15), 2718–2727.

Kotte, O., Volkmer, B., Radzikowski, J.L. & Heinemann, M. (2014) Phenotypic bistability in Escherichia coli’s central carbon metabolism. *Molecular Systems Biology*. 10 (7).

Kotte, O., Zaugg, J.B. & Heinemann, M. (2010) Bacterial adaptation through distributed sensing of metabolic fluxes. *Molecular Systems Biology*. 6 (1), 355.

Li, G.-W., Burkhardt, D., Gross, C. & Weissman, J.S. (2014) Quantifying Absolute Protein Synthesis Rates Reveals Principles Underlying Allocation of Cellular Resources. *Cell*. 157 (3), 624–635.

Moreno-Gámez, S., Kiviet, D.J., Vulin, C., Schlegel, S., et al. (2020) Wide lag time distributions break a trade-off between reproduction and survival in bacteria. *Proceedings of the National Academy of Sciences*.

Moxon, R. & Kussell, E. (2017) The impact of bottlenecks on microbial survival, adaptation, and phenotypic switching in host–pathogen interactions. *Evolution*. 71 (12), 2803–2816.

Rocabert, C., Beslon, G., Knibbe, C. & Bernard, S. (2020) Phenotypic Noise and the Cost of Complexity. *bioRxiv*. 2020.02.26.963843.

Schreiber, F., Littmann, S., Lavik, G., Escrig, S., et al. (2016) Phenotypic heterogeneity driven by nutrient limitation promotes growth in fluctuating environments. *Nature Microbiology*. 1 (6), 16055.

Taniguchi, Y., Choi, P.J., Li, G.-W., Chen, H., et al. (2010) Quantifying E. coli Proteome and Transcriptome with Single-Molecule Sensitivity in Single Cells. *Science*. 329 (5991), 533–538.

Zhang, Z., Qian, W. & Zhang, J. (2009) Positive selection for elevated gene expression noise in yeast. *Molecular Systems Biology*. 5 (1), 299.
